# New lineages of photobionts in Bolivian lichens expand our knowledge on habitat preferences and distribution of *Asterochloris* algae

**DOI:** 10.1038/s41598-021-88110-0

**Published:** 2021-04-22

**Authors:** Magdalena Kosecka, Beata Guzow-Krzemińska, Ivana Černajová, Pavel Škaloud, Agnieszka Jabłońska, Martin Kukwa

**Affiliations:** 1 Department of Plant Taxonomy and Nature Conservation, Faculty of Biology, University of Gdańsk, Wita Stwosza 59, 80308 Gdańsk, Poland; 2 Faculty of Science, Department of Botany, Charles University, Benatska 2, 12801 Praha 2, Czech Republic

**Keywords:** Biodiversity, Molecular ecology, Genetic markers

## Abstract

We studied the biodiversity of *Asterochloris* photobionts found in Bolivian lichens to better understand their global spatial distribution and adaptation strategies in the context of a worldwide phylogeny of the genus. Based on nuclear ITS rDNA, the chloroplast *rbc*L gene and the *actin* type I gene we reconstructed a phylogenetic tree that recovered nine new *Asterochloris* lineages, while 32 Bolivian photobiont samples were assigned to 12 previously recognized *Asterochloris* lineages. We also show that some previously discovered *Asterochloris* photobiont species and lineages may occur in a broader spectrum of climatic conditions, and mycobiont species and photobionts may show different preferences along an altitude gradient. To reveal general patterns of of mycobiont specificity towards the photobiont in *Asterochloris*, we tested the influence of climate, altitude, geographical distance and effects of symbiotic partner (mycobiont) at the species level of three genera of lichen forming fungi: *Stereocaulon*, *Cladonia* and *Lepraria*. Further, we compared the specificity of mycobionts towards *Asterochloris* photobionts in cosmopolitan, Neotropical, and Pantropical lichen forming fungi. Interestingly, cosmopolitan species showed the lowest specificity to their photobionts, but also the lowest haplotype diversity. Neotropical and Paleotropical mycobionts, however, were more specific.

## Introduction

Lichens are a marvelous example of ubiquitous, symbiotic fungi. Their thalli contain eukaryotic green algae and/or cyanobacteria, which represent the photosynthetic partners and are called photobionts; however, numerous bacteria and fungi also occur in the lichen symbiosis^1–3^. Many lichens are widely distributed, but it seems that photobionts in lichen symbioses may show their own habitat preferences independent of the lichenized fungus itself^4–6^. Since the organisms’ environmental preferences may be closely related to their distribution, the geographical patterns of the photobionts may be different from their fungal partners^5^. Some phylogenetic lineages, species or OTUs (operational taxonomic units), of photobionts are globally distributed, but many photobionts have only been recorded in a specific region or habitat^5,7–9^. In many cases, due to the uneven sampling of the lichen symbionts tested, it is too early to precisely define biogeographic patterns for lichenized algae; this especially applies to photobionts from the tropics^5,7^.

*Asterochlori*s is one of the most common photobionts in lichen symbioses, but it is mostly restricted to certain phylogenetic lichen groups, of which undoubtedly the best sampled and studied are these associated with members of *Cladonia*, *Lepraria* and *Stereocaulon*^4,5,9–18^*.* So far, eighteen species have been identified within *Asterochloris*^13,14,19–22^. Nevertheless, recent research^5,9,14,22,23^ demonstrates there are many phylogenetic *Asterochloris* lineages that represent still unrecognized species, which, apart from differences in the molecular markers, also differ in climate and substrate preferences. Moreover, Škaloud et al.^14^ indicated that genetic diversity, ecology, biogeography and specificity to mycobiont partners should all be taken into account in species delimitation. In addition, it has been revealed that some lichens usually associated with *Asterochloris* spp. may have additional photobionts in their thalli, e.g., *Chloroidium*^9^ or *Vulcanochloris*^9,16^.

To date, many analyses have been carried out to determine the distribution patterns of lichenized algae, and many authors name climate as the most important factor shaping their distribution^4,5,24–26^. In addition, photobiont lineages specific to a particular geographic region have been reported^24,25^, but the exact factors driving photobiont selection remain unexplored. Some studies have indicated that the mycobiont can be highly specific to its photobiont^7,23,27–31^; however, mycobiont generalists (sensu Yahr et al.^32^, i.e. with low photobiont specificity) are also common, especially in lichens that have wide geographical and ecological ranges^33–35^. In general, photobiont selection is driven by phylogenetic specialization, mycobiont reproductive strategy, availability of motile and airborne photobiont cells, as well as key ecological factors for both symbionts^32,33,36–40^.

Despite extensive research efforts, there are many OTUs that are still unnamed. Few studies have been performed using the material from the Neotropics^23,41^, and from this region most *Asterochloris* photobionts are of unknown taxonomic affiliation^9,12,23^. In the present study we focused on *Asterochloris* biodiversity in lichen symbioses in Bolivian Andean vegetation to better understand their global spatial distribution.

Bolivia represents the geographical and geological synthesis of South America its geology is a cross-section of all geological eras^42^. The eastern Andean foothills have been identified as the most biologically diverse region of the country. Humidity, rainfall and temperature are important for many biological processes that led to a high speciation degree and endemism. The Yungas forests are considered the center of diversity for plants dependent on the humid seasonal climate^42^; this is potentially true for lichens as well. This makes Yungas forests the most important center of endemism in Bolivia^42,43^.

The main aim of this work is to examine the genetic diversity of *Asterochloris* photobionts in lichens from Bolivia and to reconstruct their phylogenetic relationships. Additionally, to better understand global spatial distribution and adaptation strategies of *Asterochloris* algae, we studied the role of various factors: mycobiont host, altitude, climatic conditions, and location. For some lineages, we also assessed the level of selectivity of bionts. Additionally, we present the first record of *Vulcanochloris* from the Neotropics.

## Results

### Phylogenetic analyses

**Figure 1:**
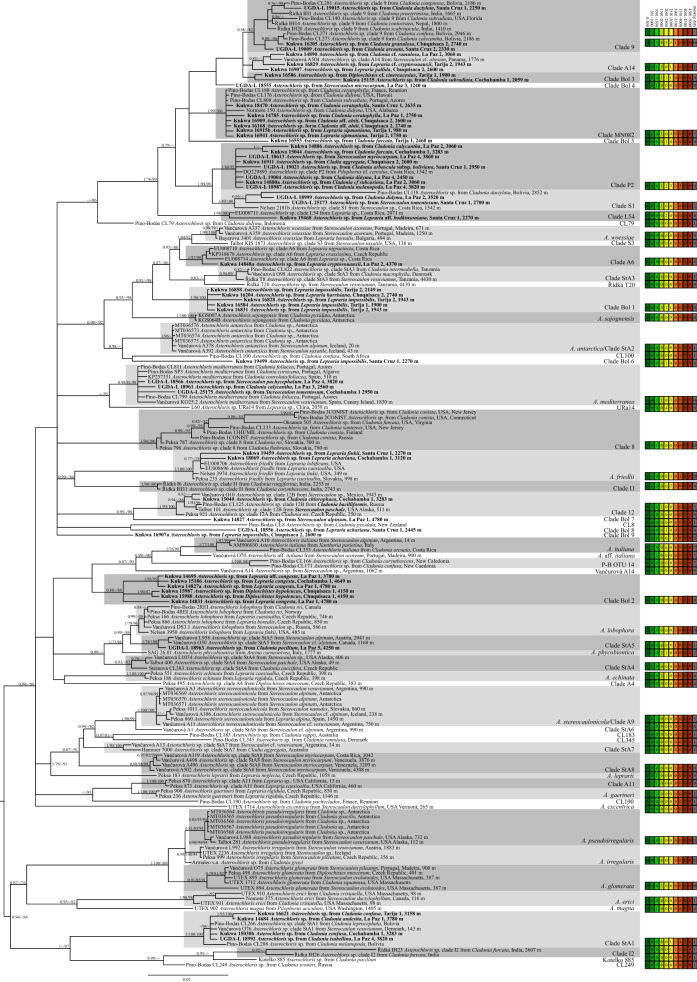
Majority-rule consensus tree from Bayesian analysis of *Asterochloris* based on ITS rDNA, *rbc*L and *actin* type I locus data set with posterior probabilities and bootstrap support values from RaxML and *IQ-TREE* analysis presented near the branches. For each record GenBank accession no. or voucher no. (for newly sequenced samples) are followed with photobiont name (if known), their mycobiont host name and the origin of specimen together with altitude (if known). Newly sequenced photobionts from Bolivia are marked in bold. Species or phylogenetic lineages are marked in boxes with appropriate names. Based on altitude of the samples, which belongs to particular phylogenetic lineage, we added summary of abundance for each lineage. Samples from 0 to 500 m a.s.l. are in first box, from 501 to 1000 are in second box, from 1001 to 1500 are in third box, from 1501 to 2000 are in fourth box, from 2001 to 2500 are in fifth box, from 2501 to 3000 are in sixth box, from 3001 to 3500 are in seventh box, from 3501 to 4000 are in eighth box, from 4001 to 4500 are in ninth box, from 4501 to 5000 are in tenth box and samples with missing data of altitude are in eleventh box, marked in grey.

In the present study we generated 54 new ITS rDNA sequences, 29 sequences of the chloroplast *rb*cL gene fragment, and 14 of the *actin* type I of *Asterochloris* algae. In addition, we obtained one ITS rDNA sequence of genus *Vulcanochloris* (Table S1). The Bayesian phylogenetic tree inferred the genus *Asterochloris* to be divided into two major clades (Fig. 1), similarly to the results of Vančurová et al.^17^. The first major clade (PP = 0.94, ML = –/84) consists of four poorly supported subclades and a single lineage of *A. excentrica*, although in Vančurová et al. only two subclades were found^17^. The second clade (PP = 0.94, ML = –/84) consists of two subclades. Among them, five lineages represent previously described species: *Asterochloris erici, A. glomerata, A. irregularis, A. magna* and *A. pseudoirregularis*; an additional four undescribed lineages also occur in this clade. Altogether we recovered 61 lineages (Fig. 1), 52 of which have been previously identified^4,5,9,11–13,15,16,22,23^, and for those we applied the same nomenclature as in previous papers. 32 *Asterochloris* samples obtained from Bolivian specimens grouped within 12 of these previously recognized clades, of which only two lineages were ascribed to the species level^14,20^ (Fig. 1). The remaining nine photobiont lineages (marked as Bol 1–9) consisted only of Bolivian samples and formed new lineages of *Asterochloris*, probably representing undescribed species, as shown in the ABGD analysis.

Furthermore, in 12 localities from Bolivia presented in this study and two previously reported additional Bolivian localities^23^, we found diverse *Asterochloris* lineages. In locality Chuquisaca 2 (N = 7; for locality codes see Table S1), we found six different *Asterochloris* photobionts; two were detected for the first time. Additionally, each species of *Cladonia* and *Lepraria* from this locality was found to be associated with a different *Asterochloris* lineage. In locality Cochabamba 1 (N = 7), we identified seven photobionts lineages: *A. mediterranea*, *A. friedlii*, *Asterochloris* spp. StA1, clades 12, P2, Bol2 and Bol3, each associated with a different lichen species. Furthermore, we identified the same ITS rDNA haplotype belonging to *Asterochloris* sp. clade Bol1 in *Lepraria impossibilis* in a range of altitudes from 1900 to 2149 m a.s.l. (Table S14). Similarly, photobionts belonging to clade Bol2 from *L. congesta* and *L*. aff. *congesta* were collected from 3780 to 4780 m a.s.l. *Asterochloris* sp. clade MN082 was recorded in localities Chuquisaca 2, La Paz 1, Santa Cruz 1, Tarija 1 and 2, within thalli of Neotropical (*Cladonia* aff. *ahtii*, *C. ceratophylla*) or Pantropical (*Lepraria sipmaniana*) lichens in a range of altitudes from 980 to 2750 m a.s.l., while *Asterochloris* sp. clade Bol1 associates with *Lepraria impossibilis* from localities Tarija 1 and 2, and with *L. harrisiana* from locality Chuquisaca 2. *Asterochloris* sp. clade P2 was found in *Pilophorus* cf. *cereolus* from Costa Rica, and also in eight Bolivian localities in altitudes between 2237 and 3860 m a.s.l. in *Cladonia* spp., *Cladia aggregata* and *Stereocaulon myriocarpum* (Fig. 1; Tables S1, S2).

We also observed dissimilarities in photobionts composition within four habitat types: lower montane cloud forest, upper montane cloud forest section 1, upper montane cloud forest section 2 and open high Andean vegetation (Figs. S6, S7). For example, in lower montane forest (N = 12), *Asterochloris* spp. clades Bol1, Bol3, Bol4, A14, 9 and MN082 were present, while in the first section of upper montane cloud forest (N = 26), *Asterochloris* spp. clades 9, A14, L54, P2, S1, MN082 and Bol1, Bol5, Bol6, Bol8 and Bol9, *A. mediterranea* and *A. friedlii* occurred. In addition, in the second section of upper montane cloud forest (N = 28), *Asterochloris* spp. clades 9, P2, S1, 12, A14, StA1, Bol2, *A. medirerranea*, *A. friedlii* and *Vulcanochloris* sp. were detected, while in the open high Andean vegetation (N = 11), *Asterochloris* spp. clades 8, StA1, StA5, A6, Bol2 and Bol7 were present. This shows that diverse photobiont types may occur within a steep gradient of altitude. With increasing altitude above sea level, the average temperature and amount of precipitation diminish (Fig. S7). This means that photobionts in the open high Andean vegetation should be resistant to low temperatures and periods of drought, and species of photobionts in the lower montane cloud forest should be resistant to higher temperatures and its seasonal changes. Humidity (as seasonality of rainfall) in *Stereocaulon* has already been indicated as a determining factor for *Asterochloris*, *Vulcanochloris* and *Chloroidium*^9^.

**Figure 2:**
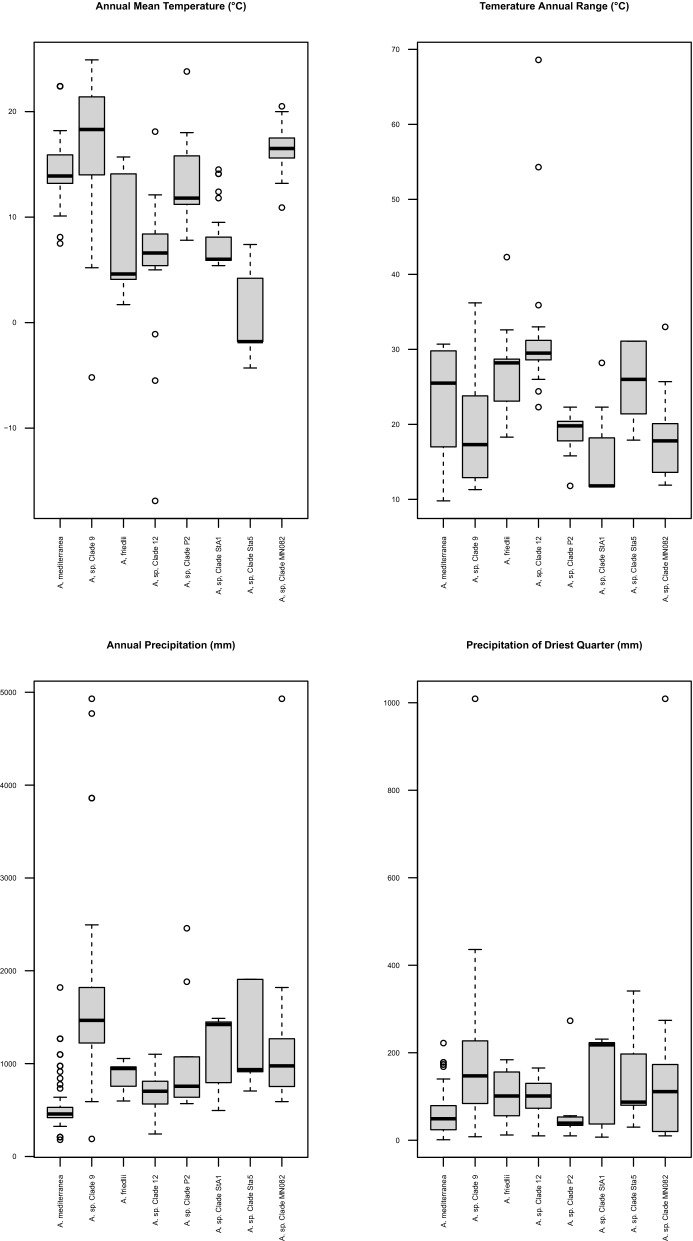
Box-plot diagram representing differences in climatic preferences for selected species and lineages of *Asterochloris* photobionts. Climatic data were obtained from the Global Climate Data—WorldClim. BIO1** = **Annual Mean Temperature (°C), BIO7 = Temperature Annual Range (°C), BIO12 = Annual Precipitation (mm), BIO17 = Precipitation of Driest Quarter (mm).

Based on the observed differences in composition of photobionts of four habitat types in Bolivia, we concluded that some previously known *Asterochloris* lineages may occur in a broader spectrum of climatic conditions (data summarized in Table S9 and in Fig. 1 in reference to altitude). Thus, *A. mediterranea* only known from the Mediterranean, temperate Europe and islands of the North Atlantic and Indian Oceans can also occur in the Neotropics. Annual precipitation in areas in which this species occurs ranges between 18 and 974 mm, with precipitation in the driest quarter between 2 and 95 mm (Fig. 2). *Asterochloris* sp. OTU25^23^ was found on islands of the North Atlantic, Indian and Pacific Oceans. We also identified this particular lineage in Bolivia in *Cladonia ceratophylla*, *C*. aff. *ahtii* and *Lepraria sipmaniana.* Our phylogenetic analysis revealed that this lineage is similar to a photobiont from *Cladonia didyma* (clade MN082) from USA^31^ and we conclude it may possess tolerance to a wide range of climatic condition. We do not have complete data for all specimens, but our rough estimate for annual precipitation for this lineage ranges between 591 and 4930 mm, with precipitation of the driest quarter between 11 and 1009 mm. *Asterochloris friedlii* was previously known from temperate Europe and USA as well as tropical areas of China and Korea^5,12^, and we found it in a Bolivian sample of the widespread *Lepraria finkii*. A similar situation was observed for *Asterochloris* sp. clade StA5, which to date has been found to associate with *Stereocaulon* spp.^9^. Likewise, *Asterochloris* sp. clade 9 found in South America (this study), India and Southern North America is associated only with *Cladonia* spp.^5,18,23,32,41^; based on our analysis, this lineage shows tolerance for a broad annual mean temperature range (− 5.2–24.9 °C) and is characterized by drought resistance and rehydration (annual precipitation: 591–4930 mm, precipitation of driest quarter: 8–1009 mm, precipitation of wettest quarter: 143–3968 mm). The widespread lineage *Asterochloris* sp. clade 12 (also found in this study) associates with *Cladonia* spp. and *Stereocaulon* spp.^5,9,11,13,23^ and is characterized by the widest annual temperature range we have seen: − 16.9–18.1 °C and high drought resistance (precipitation of driest quarter: 10–165 mm). *Asterochloris* sp. clade P2 was found to associate with different lichen species and genera (*Cladia*, *Cladonia*, *Pilophorus* and *Stereocaulon*) [this study^11,23^]. This lineage, together with closely related *Asterochloris* spp. clades L54 and S1, were all found only in the Neotropics [this study^11,23^]. So far, *Asterochloris* spp. clades L54 and A6 have been found to form a symbiosis exclusively with *Lepraria* spp. (Fig. 1) [this study^4,11^].

### Statistical analyses

To identify the impact of selected factors on the distribution of *Asterochloris* photobionts, we performed variation partitioning analyses (Tables S7, S8) that showed that our selected variables explained 46–47% of the variation for Bolivian samples. 25–26% of the variation was explained by the species of the mycobiont (Table S7), which shows low correlation between photobiont distribution and mycobiont hosts. 22–23% was explained by environmental factors. 5% of the variability was shared between mycobiont host, climate and altitude or habitat type, while, 6% of the variability was explained by climate as independent factor.

In the case of *Stereocaulon*, 39% of the variation was explained by our selected variables. The largest part of the variation in diversity of photobionts was explained by the mycobiont hosts (12%, Table S7). As independent factors, altitude explained 8% of the variation, while geographical distance—explained 4%. For *Cladonia* spp., 58% of the variability was explained by our selected variables (Table S7). 31% was explained by mycobiont species, whereas climate and geographical distance explained 5% and 3%, respectively. However, ecological factors shared 11% of the variability. In the case of *Lepraria* spp., altitude appeared to be insignificant (Table S6). Results of PCoA analyses for *Lepraria* spp. show moderate correlation between photobiont distribution and mycobiont host (35% of the variation explained by mycobiont). The remaining factors did not exert significant influence.

In addition, we selected 12 cosmopolitan, 17 Neotropical and ten Pantropical species from our dataset, and supplemented by data available in GenBank (Table S5). In the case of cosmopolitan species, variation partitioning analyses revealed that 0–13% of the variability was explained by mycobiont hosts (Table S8), climate accounted 15% of the variability; altitude and substrate appeared to be insignificant (dbRDA analyses; Table S6). Overall, 59% of the variability of *Asterochloris* distribution in Neotropical and Pantropical lichens can be explained by the mycobiont hosts. We performed PCA analyses to identify which photobionts were represented by selected groups of lichens, as a function of climate (Fig. S2). PCA ordination of all analyzed species resulted in 72.7% cumulative variance explained on the first 2 axes (first—47.8%, second—24.9%). Lichen groups and their photobionts, respectively, showed indifference relative to climatic parameters: i.e., they are scattered across the hyperdimensional climatic space (Fig. S2). However, cosmopolitan, Neotropical and Pantropical species as groups represent different range of climatic conditions. In the case of cosmopolitan species, the total explained variation in the biplot was 68.4% by component 1 and 2 (Fig. S3). PCA ordination of Neotropical species, on the other hand resulted in 84.6% (Fig. S4) cumulative variance explained on the first 2 axes; and for Pantropical species it was 78.7% (Fig. S5).

However, in cosmopolitan, Neotropical and Pantropical groups of lichens (and also between them), different genera show indifferences, dependent on diverse climatic conditions (potentially climatic preferences). Tropical species prefer higher temperatures in warm and wet seasons, while the cosmopolitan species are adapted to extreme temperatures (BIO4, 7).

Due to the dissimilarities we observed in the photobionts composition of the four habitat types in Bolivia, we performed PCA to visualize variability of photobionts of those habitat types in the context of climatic factors (Fig. S6). This shows that different conditions prevail in each habitat, however, the ranges of photobionts from different habitats may overlap.

### Haplotypes

Due to differences in the impact of the mycobiont species on the distribution of photobionts in selected genera of lichens, as well as differences in tolerance for climatic conditions, we performed ITS rDNA haplotype analyses to study the associations of mycobiont hosts and photobionts in selected groups of lichens. In the case of *Asterochloris* photobionts from 200 *Stereocaulon* samples (Table S10), we found low haplotype diversity (0.28) and detected 55 photobiont ITS rDNA haplotypes (Tables S10, S16), of which 24 were identified in samples from tropics, 36 in samples from the temperate zone, and 5 from both regions. However, haplotype diversity for tropical samples appeared to be higher (0.52) than that from temperate areas (0.24) (Table S17). *Asterochloris irregularis* appeared to be the most common photobiont associated with *Stereocaulon* spp. in temperate climates (62 samples). In *Stereocaulon* samples from Bolivia, we found five distinct haplotypes representing *A. mediterranea*, *Asterochloris* spp. clades P2, S1, and Bol4 and Bol7. Moreover, some species showed a high flexibility in their photobiont choice, and furthermore, it appears their photobiont pools may differ depending on the climate, e.g. in *Stereocaulon vesuvianum* we found haplotypes belonging to *Asterochloris glomerata, A. irregularis, A. pseudoirregularis, Asterochloris* spp. clades A9, StA1 and StA7 in temperate areas, while *Asterochloris* spp. clades StA3, StA5, T20 and *A. mediterranea* (Table S10) in tropical regions.

In the case of *Asterochloris* associated with *Cladonia* spp., we found 268 ITS rDNA haplotypes (haplotype diversity = 0.42) based on 630 samples (Tables S11, S16). 132 originated from tropical regions, 143 from temperate areas and only 10 *Asterochloris* haplotypes were noted in both regions. Moreover, we observed differences in photobiont haplotype diversity within the tropical and temperate *Cladonia* samples (tropical—0.63, temperate—0.35). The most common photobiont in *Cladonia* spp. from tropical areas seems to be *Asterochloris* sp. clade 9 (71 samples); this potentially new species is represented by 54 haplotypes in our dataset. Moreover, samples of this clade were also found in 13 *Cladonia* specimens from temperate regions. Bolivian specimens of *Cladonia* spp. were found to associate with 34 haplotypes belonging to *A. mediterranea*, *Asterochloris* spp. clades 9, 12, A14, S1, StA1, StA5, P2, MN082, Bol3 and Bol5.

Photobionts from *Lepraria* spp. (Tables S12, S16), represented by 44 haplotypes of *Asterochloris,* showed high haplotype diversity (0.54). In tropical climates 14 haplotypes were found, while in temperate areas we found 30 haplotypes. The most common photobiont in *Lepraria* spp. is a haplotype of *A. friedlii* (14 sequences from both regions). Moreover, haplotype diversity was higher in tropical (0.70) than temperate (0.49) regions (Table S17).

In the case of lichens representing cosmopolitan distribution patterns, we observed 62 *Asterochloris* haplotypes (Table S13) with high haplotype diversity (0.53) (Table S16). These haplotypes belong to 30 *Asterochloris* lineages. Samples from tropical climates were found to associate with 14 lineages, while specimens from temperate climates associated with 21. Only one was found in both regions (haplotype 28 in Table S13). Some lichen species appear to adopt different *Asterochloris* haplotypes in different climatic regions, e.g. *Cladonia furcata* associates with *Asterochloris* spp. clades 12 or 8 in temperate areas, while other haplotypes were found in tropical areas (clades I1 and I2 in India, and clades P2 and Bol5 in Bolivia). Remarkably, *Stereocaulon alpinum* appears to associate with at least 11 different *Asterochloris* lineages, of which clade Bol7 and *A. lobophora* have only been found in tropical climates. Interestingly, *Asterochloris* sp. clade StA5 was found both in tropical and temperate regions: Bolivia, Austria (one haplotype) and in Canada (a different haplotype). Additionally, *Stereocaulon alpinum* collected in temperate regions was also found to associate with *A. irregularis*, *A. italiana, A. phycobiontica, A. woessiae* and *Asterochloris* spp. clades StA2, StA4 and A9 (Table S10). In the case of *Cladonia pyxidata,* it was found to associate with haplotypes of *A. glomerata, A. lobophora,* and *Asterochloris* sp. clade12 in temperate regions; with *A. sejongensis* in Antarctica; and with *Asterochloris* spp. clades 12 and I1 in tropical regions. Indeed, *Asterochloris* sp. clade 12 may associate with this species in both regions (Table S11). Seventeen Neotropical lichen species were analyzed; among them we identified 29 haplotypes of *Asterochloris* belonging to 16 lineages (Table S14) with high haplotype diversity (0.83) (Table S16). In the case of the Pantropical group, ten lichen species associated with 32 *Asterochlori*s haplotypes belonging to 13 lineages (Table S15) and showed high haplotype diversity (0.80) (Table S16). Within Neotropical and Pantropical lichens, we found multiple *Asterochloris* haplotypes either restricted to a single species or with potentially wide selectivity (i.e., occurring in different species in similar or diverse localities).

## Discussion

Little is still known about the nature of the associations between mycobionts and photobionts. Only several works have explored the diversity of *Asterochloris* photobionts in the tropics^5,9,11,12,18,31,32,41^. In this study, we recovered nine new *Asterochloris* lineages, while 29 Bolivian photobiont samples were assigned to 12 previously recognized *Asterochloris* lineages, from which only two have been so far formally described (Fig. 1).

In this study we also showed that some previously known *Asterochloris* photobiont lineages may occur in a broader spectrum of climatic conditions, e.g. *A. mediterranea* is here reported for the first time from Neotropics. On the other hand, alpine and psychrophilic *Asterochloris* sp. clade StA5^9^ may occur in open high Andean vegetation and may show higher drought resistance (Precipitation of Driest Quarter for sample UGDA-L 18963 = 30 mm) than previously thought (80–341 mm) (Fig. 2). *Asterochloris* spp. clades P2, A14 and L54 are considered Neotropical lineages, as well as clades Bol1 and Bol2 recovered in this study, neither of which is closely related to previously described lineages.

Preferences of mycobionts for certain types of photobionts have been repeatedly recognized for *Trebouxia*^8,29,44^, *Asterochloris*^9,14^, or Trentepohliaceae^7^. The photobiont’s ecological specialization probably determines the mycobiont’s selection for its symbiont^4,44,45^. Furthermore, horizontal transmission has probably increased the taxonomic range of compatible photobiont partners^15^; for example Vančurová et al.^9^ showed that several algal species or lineages show specificity towards a single mycobiont species. On the other hand, they also recovered lineages that were not specific towards a single mycobiont. Our data shows that previously described *Asterochloris* lineages can associate with broader ranges of mycobionts. For example, *Asterochloris* spp. clades StA1 and StA5, were previously found only within *Stereocaulon* spp., but we found them associated with different *Cladonia* species, suggesting a lower level of selectivity and specificity (sensu Beck et al.^10^). Interestingly, *Asterochloris* sp. clade MN082 was found in samples of *L. sipmaniana*, *Cladonia* aff. *ahtii,* and *C. ceratophylla* from different localities ranging in altitude from 980 to 2750 m a.s.l; these results are preliminary, however, further analyses are necessary to determine the exact selectivity level of these algae.

Ecological factors may modulate the availability of photobiont species/strains at various sites, thus reducing the number of possible associations in isolated populations. The influence of altitude gradient on photobiont population structure was previously described^7,46–48^. In our study, we found some changes in photobiont populations along altitude gradients; e.g., *Asterochloris* spp*.* clades Bol1 and MN082 were present in lower montane cloud forest and upper montane cloud forest section 1; in upper montane cloud forest section 1 and section 2 were *Asterochloris* spp. clade P2 and S1, as well as *A*. *mediterranea* and *A. friedlii*. In each altitude dependent habitat, unique *Asterochloris* lineages occur. We need more data to define exact ecological niche for those lineages.

To reveal global patterns of specificity of the mycobionts towards the photobiont in *Asterochloris*, we tested the influence of climate, altitude, geographical distance and effects of the symbiotic partner (mycobiont) at the species level for three genera of lichen forming fungi: *Stereocaulon*, *Cladonia* and *Lepraria*. *Stereocaulon* species are widespread and have broad ecological requirements^9^, and associate with many *Asterochloris* species [^14^, this study], but also *Chloroidium*^9,10^ and *Vulcanochloris* [^16^, this study]. The diversity of photobionts in *Stereocaulon* and the association between their diversity and environmental conditions was previously conducted on a global scale by Vančurová et al.^16^. The photobionts distribution in *Stereocaulon* species found in that study appeared to follow a pattern that was highly influenced by substrate type. However, in that study this was mostly evident in *Chloroidium* and *Vulcanochloris*. Our analyses confirmed the hypothesis that photobiont variability may be dependent on geographical distance^32^. In the case of *Stereocaulon*^9^, 4% of the photobiont variability was explained by a net effect of geographical distance. Furthermore, the distribution of photobiont diversity in *Stereocaulon* may also be affected by altitude (8%). It has been previously shown that the majority of *Stereocaulon* mycobiont species appear to be specific towards phycobionts^9,17^. In our study, *Stereocaulon* displayed a low level of selectivity toward photobiont lineages (Table S10). Furthermore, we report low haplotype diversity for *Asterochloris* photobionts of *Stereocaulon* that can be correlated with oversampled photobionts from temperate regions.

Yahr et al.^32^ demonstrated that geographic position and habitat are the best predictors of algal genotype distribution in *Cladonia*. Steinová et al.^15^ showed that photobionts are significantly structured by climate and geography, but the mode of reproduction was revealed to have the greatest impact on *Cladonia* photobiont diversity. In our analyses we found that 36% of the variability was explained by mycobiont at the species level. Furthermore, we report higher haplotype diversity in photobionts from *Cladonia* spp. than *Stereocaulon* spp., in which the predominating photobiont species was *Asterochloris glomerata* (21 haplotypes represented by 104 sequences; the most common haplotype represented by 51 sequences). Nonetheless, *Cladonia* and *Lepraria* are known to associate with a wide range of *Asterochloris* species^4,14^. With regards to *Lepraria,* we found that these mycobiont species associated with particular *Asterochloris* taxa from lineages B and C (sensu Vančurová, et al.^9^) and (like *Cladonia*), show moderate selectivity toward their photobionts, with a moderate influence of mycobiont species and a very low influence of climate and spatial structure. In addition, species of both genera may show different ranges of adaptation strategies. These results support a hypothesis that mycobionts can associate with numerous symbiotic partners in the case of ubiquitous lichen species adapted to various ecological conditions^8,13,23,24^. The dominant species of *Asterochloris* found within *Lepraria* spp. was *A. friedlii* (found in 22 samples, 27% of tested samples). However, more research is needed to reveal the most common *Asterochloris* spp. in *Lepraria* as well as to fully explain the factors affecting photobiont distribution.

Lichens exhibit various distribution patterns at the micro- and macro-levels. In the case of lichen-forming fungi, 16 main biogeographic patterns were distinguished (including cosmopolitan, bipolar, paleotropical, Neotropical, Pantropical, Mediterranean)^49^, but the biogeography of lichen photobionts is still poorly known. Preferential associations with locally adapted symbionts have been reported repeatedly^8,50,51^. It was hypothesized that low specificity of the host towards its symbiotic partner helps the host to take advantage of the locally adapted symbiotic partners and colonize broader geographic areas^8^. In the case of higher specificity, the host is expected to have a narrower ecological niche, and a restricted geographical distribution. In the case of lichen symbioses, the generalist pattern is more common^8,24,47^; however, a specialist pattern has been reported for *Nostoc*-associated lichen fungi^52^. We compared the specificity of mycobionts towards *Asterochloris* photobionts in three groups of lichens: cosmopolitan, Neotropical, and Pantropical. Interestingly, cosmopolitan species repeatedly showed the lowest specificity towards photobionts, but also had the lowest haplotype photobiont diversity. More haplotypes were identified in temperate regions; however, the haplotype diversity of tropical photobiont lineages was higher. This may indicate a significant under-sampling of tropical regions. In addition, the distribution of the diversity of photobionts within cosmopolitan lichens was influenced by a moderate impact of climatic conditions (15%), while in the case of Neotropical and Pantropical lichens there was a correlation between the mycobiont species and climatic conditions. This indicates the selection of locally adapted photobionts in cosmopolitan lichens, while tropical species mostly demonstrate habitat preferences. However, in the light of the results obtained here and in previous research^8,53^, we assume that the selection of a symbiotic partner may be based on the search of the best-suited photobiont. To obtain more accurate results, additional data on one species commonly found in the Neotropical and Pantropical regions are needed, with emphasis on the impact of habitat conditions.

In conclusion, to further investigate these questions, we suggest that a suitable species, representative of each fungal genus associating with *Asterochloris*, should be selected for future studies before sampling. Furthermore, to avoid geographical gaps, sampling should be extended to the whole world. In addition, on the basis of the results obtained here, it can be assumed that Bolivia, due to its geological and ecological diversity, may reflect the biodiversity of the entire Andean region in the Neotropics, where the issue of photobionts' biodiversity still remains unresolved. Indeed, the tropics may be an important, under-explored source of hidden photobionts biodiversity.

## Materials and methods

### Material

54 lichens samples representing 4 genera: *Cladoni*a, *Diploschistes*, *Lepraria* and *Stereocaulon* containing *Asterochloris* photobionts from various habitats in Bolivia were selected randomly for studying the biodiversity of Bolivian *Asterochloris* photobionts. Lichens were collected from various substrata (rocks, soil, tree bark, wood, and bryophytes). The majority of samples were obtained from Yungas cloud forest, but also from Tucuman-Bolivian forest and dry inter-Andean forest.

Lichen samples were collected in Bolivia with the permission of Ministerio de Media Ambiente y Agua (MMAYA/VMABCC GDF/DGBAP/MEG No 03272/2016) and in cooperation with Herbario Nacional de Bolivia (LPB), who in turn made specimens available to the herbarium of University of Gdańsk. Original samples are deposited in herbarium LPB, with duplicates stored in University of Gdańsk (UGDA). Lichen species were determined using appropriate identification keys^54–58^. Morphology, secondary chemistry, and for *Leprari*a spp., sequencing of nucITS rDNA of mycobiont^59^ were used for specimen identification. Lichen substances were identified with thin-layer chromatography (TLC) in solvents A and C^60^. Detailed locality data are presented in Supplementary Data Table S1.

The ranges of distribution were defined for lichen species used in this study—Cosmopolitan, Neotropical or Pantropical, based on the division proposed by Galloway^49^. The best sampled species were selected, and after scrutiny of the literature, a database was created for further analysis.

### Molecular methods

Well-preserved specimens lacking any visible symptoms of fungal infection were used for DNA isolation (total lichen DNA) following the CTAB protocol^61^. For molecular identification of the photobionts (*Asterochloris*), the nuclear internal transcribed spacer (ITS, ITS1-5.8S-ITS2)^11,31,62^, the chloroplast *rbc*L gene and in few cases of unique lineages of *Asterochloris*^63–65^, and the *actin* type I gene were amplified^10^. The PCR condition are presented in Supplementary Data Table S3. Sequencing was performed in Macrogen® in Amsterdam, Netherlands using amplification primers. Sequences were compared to the sequences available in GenBank using Megablast searchers^66^ to verify their identity and detect potential contaminants. The newly obtained sequences of the ITS rDNA, and fragments of the *rblc*L gene and the *actin* type I regions were deposited in GenBank (Table S1).

### Phylogenetic analyses

The *Asterochloris* sequence datasets were analyzed as a concatenated dataset of all loci. In total, 189 sequences with a total of 2618 sites were generated. The ITS rDNA dataset consisted of 188 sequences, including 54 newly obtained and 134 previously published representative sequences of taxa retrieved from GenBank. These representative sequences were selected following the latest review paper by Vančurová et al.^9^, as well as additional, new *Asterochlori*s spp. described by Kim et al.^22^ and Pino-Bodas and Stenroos^23^. To avoid confusion, the nomenclature of some mycobiont species associated with *Asterochloris* has not been updated according to the newest taxonomical works; these are: *Lepraria borealis* and *L. caesiolaba* (both now subsumed under *L. neglecta*), *L. lobificans* auct. (now *L. finkii*), *L. nigrocincta* (now *L. yunnaniana*) and *Parmelinopis minarum* (now *Hypotrachyna minarum*). The chloroplast *rbc*L gene dataset consisted of 64 sequences (29 newly obtained sequences). The *actin* type I dataset consisted of 132 sequences: 14 newly obtained sequences, and previously published sequences. Sequence alignment was automatically performed using MAFFT—Multiple Alignment using Fast Fourier Transform^67^ as implemented in UGENE^68^. Phylogenetic relationships were inferred with Bayesian Inference (BI) carried out in MrBayes v.3.2.2^69^. In cases of which *rbc*L or *actin* sequences were lacking, they were treated as missing data. Two parallel MCMC runs were performed, using four independent heated chains and 10 million generations, sampling every 1000th tree. The initial 2500 trees of each run (25%) were discarded as burn-in, and posterior probabilities were estimated by constructing a majority-rule consensus tree of all sampled post-burn-in trees. Maximum likelihood (ML) analyses were performed using RAxML-HPC v.8 with 1000 bootstrap replicates (ML-BS), the GTRGAMMAI model^70^ and the edge-linked partition model in *IQ-TREE*^71,72^ on the CIPRES Science Gateway^73^. The best-fit substitution models and block (Table S4) were selected using Akaike Information Criterion (AIC) as implemented in PartitionFinder 2^74^. The tree topology obtained by ML method did not contradict the Bayesian tree; therefore, only the Bayesian tree is shown. The consensus trees were visualized using FigTree v1.4.2^75^. Branches with bootstrap support ≥ 70% and posterior probabilities ≥ 0.95 were considered to be strongly supported. Analogically, an ITS rDNA dataset of *Vulcanochloris*, including a single new sequence of *Vulcanochloris* sp. detected in *Stereocaulon pityryzans* (UGDA-L 18522), was analyzed. It consisted of 16 sequences, of which 15 were previously published sequences were retrieved from GenBank.

### OTUs delimitation

To delimit *Asterochloris* OTUs, the Automatic Barcode Gap Discovery method (ABGD) was used, following Leavitt et al.^27^. The analyses were performed using the webserver (https://bioinfo.mnhn.fr/abi/public/abgd/abgdweb.html) separately for the ITS rDNA and actin datasets. The Jukes and Cantor (JC69) model was applied to calculate the genetic distance. Pmin = 0.001, Pmax = 0.01, step = 10, Nb bins = 20 and X (relative gap width) = 0.5 were used. Next, comparisons of the inferred OTUs were made based on both markers. Affiliation to individual species or *Asterochloris* phylogenetic lineages used in further analyses were estimated based on published data. These data are available in Table S2. In total, 61 OTUs were inferred.

### Statistical analyses

The relationship between species richness of Bolivian photobionts and lichen species, as well as the relative effects of climate, altitude, geographical distances, substrate, and habitat type, were analyzed. In the case of data obtained from GenBank, only records with precise geospatial coordinates were used. The comparative effects of selected variables were analyzed by variation partitioning in redundancy analyses, using the *varpart* function in the *vegan* package^76^. The phylogenetic distances of photobionts were used as a response variable, coded as the first 10 PCoA axes. Climatic data were obtained from the Global Climate Data**—**WorldClim Version 2^77^ at a resolution of 2.5 arc minutes. The 19 environmental variables, altitude, substrate and habitat type were transformed into principal component variables (PCs). Principal coordinates of neighbor matrices vectors (PCNM) representing the geographical distances at various spatial scales^78^ were obtained from the transformation of geographical distance values (latitude and longitude). PCNM vectors were calculated based on the pairwise geographical distances obtained by the *distGPS* function of the BoSSA package^79^. Distance-based redundancy analyses (dbRDA)^80^ were used to select statistically significant predictors for explaining variation for each of the data-sets used in the variation partitioning analyses.

Variation partitioning analyses were carried out separately for all Bolivian data obtained in this study (N = 54) and in Pino-Bodas & Stenroos^23^, and also for *Asterochloris* from selected lichen genera, i.e. *Cladonia* (N = 179), *Stereocaulon* (N = 169) and *Lepraria* (N = 34) due to better sampling of these genera. We found that the substrate influence appeared to be statistically insignificant and did not correlate with our Bolivian datasets (Table S6). Furthermore, due to multiple missing data for this particular variable, we decided to omit this factor in the analyses of the datasets of the three genera. Variation partitioning analyses were also performed for selected group of lichen forming fungi representing different distribution patterns, i.e. cosmopolitan (N = 77), Neotropical (N = 30) and Pantropical (N = 23) (patterns of distribution identified based on the literature^54–58^; Table S5). Due to limited data for species showing different distribution patterns, different PCoA analyses schemes were used. A series of analyses were performed using mycobiont host as an explanatory variable constantly together with an other variable, which was replaced in subsequent series (geographical distance, altitude, substrate and climate; Table S8). We took into account only morphospecies; we did not consider the possibility of one morphospecies comprising different phylogenetic lineages (i.e.cryptic species) a situation that can potentially be geographically or ecologically more restrictive, as has been demonstrated in the Cladoniaceae^81^.

To detect and visualize the differential ordination (tendency or strategies) of samples in the hyperspace, we performed Principal Component Analyses (PCA) according to climatic factors (BIO1–BIO19) and the grouped results depending on the distribution patterns of lichen species and the genus of mycobiont host. In addition, we performed these analyses for Bolivian samples, grouping results by habitat type. All analyses were performed in R v 3.6.0^82^, using RStudio v.1.2.1335^83^.

### Haplotypes

To visualize the precise interaction between mycobionts and photobionts, we inferred haplotypes from a pairwise character difference matrix of ITS rDNA sequences using the *haplotype* function in *haplotypes* package^84^. These analyses were carried out for six data sets (Tables S10–14) consisting of sequences newly obtained for this paper aligned with additional sequences downloaded from GenBank of *Asterochloris* from genera *Cladonia*, *Stereocaulon* and *Lepraria.* We also ran analyses for lichen-forming fungi representing different distribution patterns: cosmopolitan, Neotropical, Pantropical. Lastly, we measured nucleotide diversity, the number of haplotypes, and haplotype diversity as further estimations of differences between groups.

## Supplementary Information

Supplementary Tables.

Supplementary Figures.

## Data Availability

DNA Sequences are deposited in GenBank: ITS rDNA MW847807-MW847861, actin type I locus MW845956- MW845969, and rbcL gene MW847862- MW847890. DNA matrix used for phylogenetic analyses of Asterochloris, as a concatenated dataset of all loci is available at TreeBASE version 2 web server^85,86^, http://purl.org/phylo/treebase/phylows/study/TB2:S28062.
