# Supplementary material for: New lineages of photobionts in Bolivian lichens expand our knowledge on habitat preferences and distribution of Asterochloris algae
Source: Sci Rep. 2021 Apr 22;11:8701. doi: 10.1038/s41598-021-88110-0 (PMC8062552; doi:10.1038/s41598-021-88110-0)
Supplement: Supplementary file 2 — Supplementary Figures. [file 41598_2021_88110_MOESM2_ESM.docx]

**Supplementary Data**

New lineages of photobionts in Bolivian lichens expand our knowledge on habitat preferences and distribution of *Asterochloris* algae

Magdalena Kosecka^1^

Department of Plant Taxonomy and Nature Conservation, Faculty of Biology, University of

Gdańsk, Wita Stwosza 59, PL-80-308 Gdańsk, Poland, e-mail:

magdalena.kosecka@ug.edu.pl

Beata Guzow-Krzemińska

Department of Plant Taxonomy and Nature Conservation, Faculty of Biology, University of

Gdańsk, Wita Stwosza 59, PL-80-308 Gdańsk, Poland, e-mail: beata.guzow-krzeminska@ug.edu.pl

Ivana Černajová

Faculty of Science, Department of Botany, Charles University, Benatska 2, CZ-12801, Praha

2, Czech Republic**,** e-mail: ivkacerka@gmail.com

Pavel Škaloud

Faculty of Science, Department of Botany, Charles University, Benatska 2, CZ-12801, Praha

2, Czech Republic**,** e-mail: skaloud@natur.cuni.cz

Agnieszka Jabłońska

Department of Plant Taxonomy and Nature Conservation, Faculty of Biology, University of

Gdańsk, Wita Stwosza 59, PL-80-308 Gdańsk, Poland, e-mail: agnieszka.jablonska@ug.edu.pl

Martin Kukwa

Department of Plant Taxonomy and Nature Conservation, Faculty of Biology, University of

Gdańsk, Wita Stwosza 59, PL-80-308 Gdańsk, Poland, e-mail: martin.kukwa@ug.edu.pl

Corresponding author

Figure S1. Majority‐ rule consensus tree from Bayesian analysis of *Vulcanochloris* based on ITS rDNA data set with posterior probabilities and bootstrap support values from RaxML analysis presented near the branches. For each record GenBank accession no. or voucher no. (for newly sequenced sample) are followed with photobiont name (if known), mycobiont host name and the origin of specimen together with altitude. Newly sequenced sample from Bolivia is marked in bold.


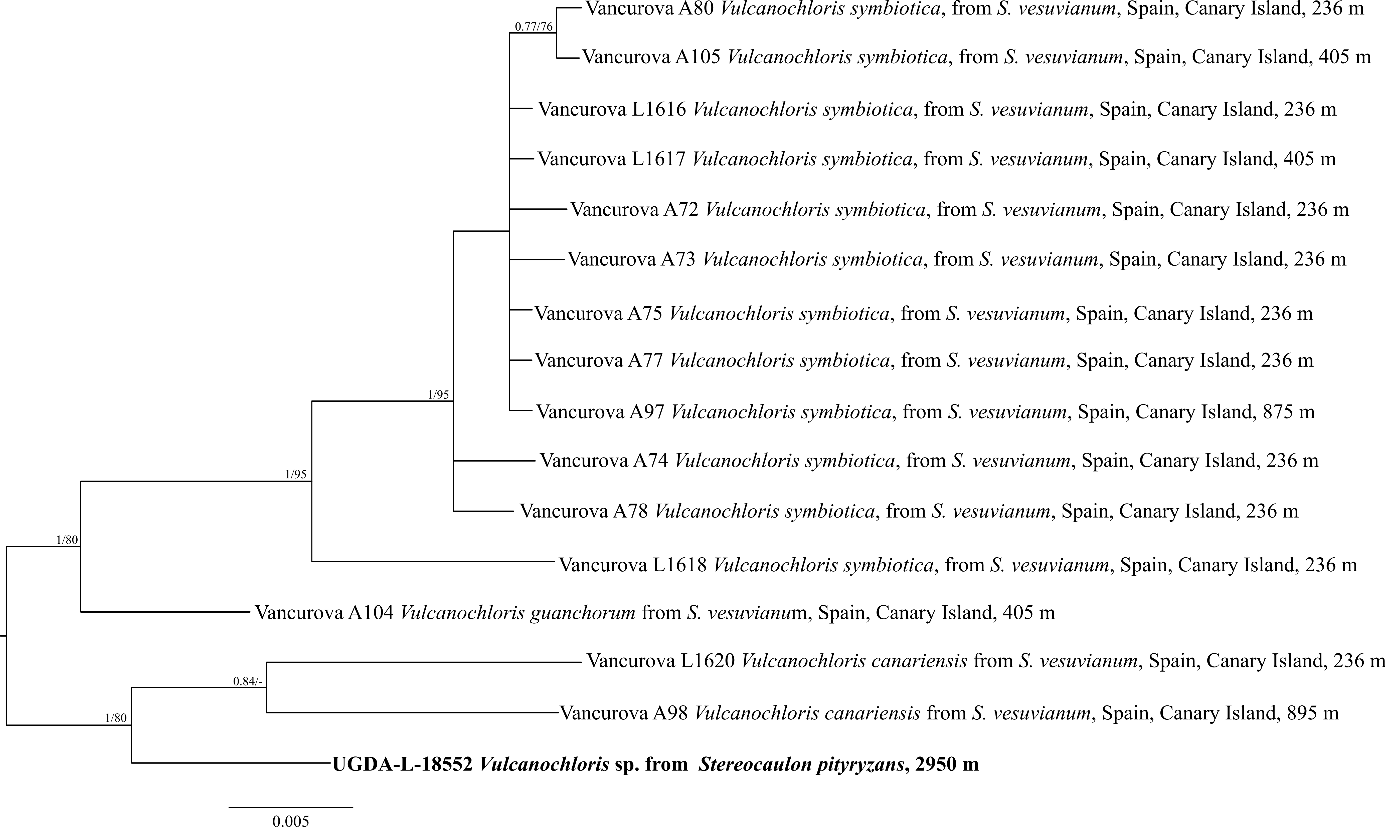


Figure S2. PCA result of *Asterochloris* distribution depending on climatic factors and distribution pattern of lichen forming fungi. Climatic data were obtained from the Global Climate Data – WorldClim. BIO1 = Annual Mean Temperature, BIO2 = Mean Diurnal Range (Mean of monthly (max temp - min temp)), BIO3 = Isothermality (BIO2/BIO7) (×100), BIO4 = Temperature Seasonality (standard deviation ×100), BIO5 = Max Temperature of Warmest Month, BIO6 = Min Temperature of Coldest Month, BIO7 = Temperature Annual Range (BIO5-BIO6), BIO8 = Mean Temperature of Wettest Quarter, BIO9 = Mean Temperature of Driest Quarter, BIO10 = Mean Temperature of Warmest Quarter, BIO11 = Mean Temperature of Coldest Quarter, BIO12 = Annual Precipitation, BIO13 = Precipitation of Wettest Month, BIO14 = Precipitation of Driest Month, BIO15 = Precipitation Seasonality (Coefficient of Variation), BIO16 = Precipitation of Wettest Quarter, BIO17 = Precipitation of Driest Quarter, BIO18 = Precipitation of Warmest Quarter, BIO19 = Precipitation of Coldest Quarter.


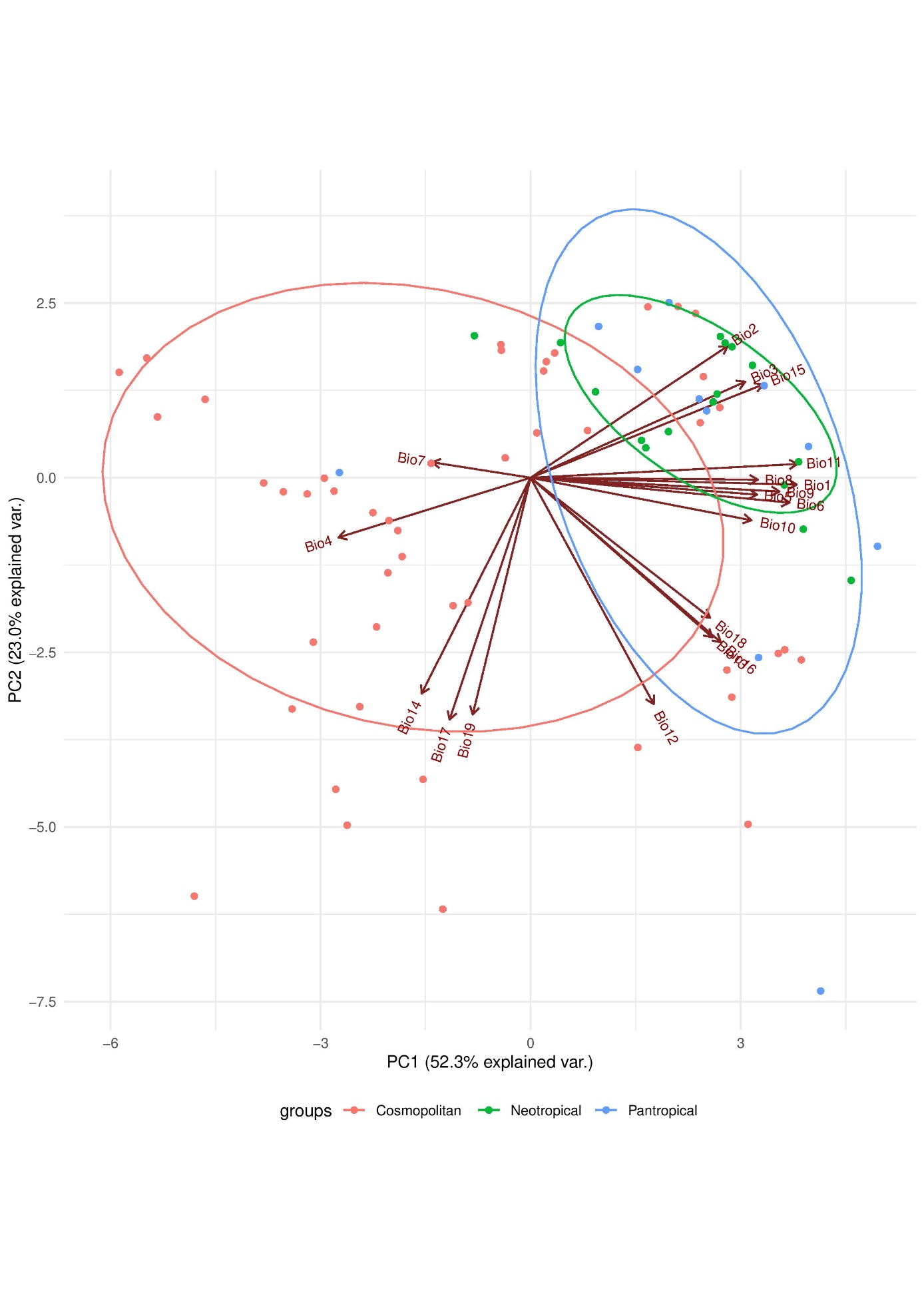


Figure S3. PCA result of *Asterochloris* distribution depending on climatic factors and taxonomic affiliation of lichen forming fungi for cosmopolitan lichens.


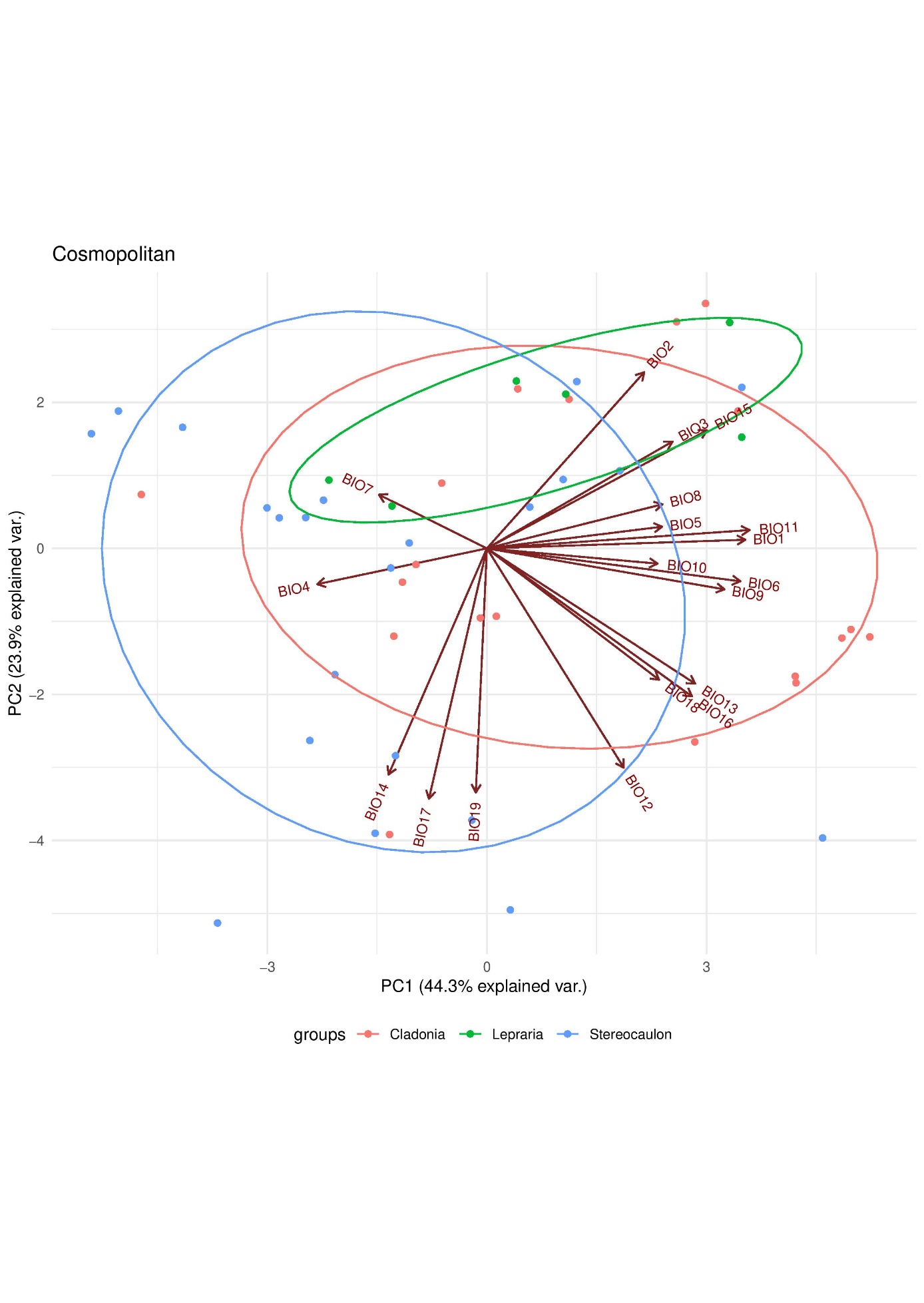


Figure S4. PCA result of *Asterochloris* distribution depending on climatic factors and taxonomic affiliation of lichen forming fungi for Neotropical lichens.


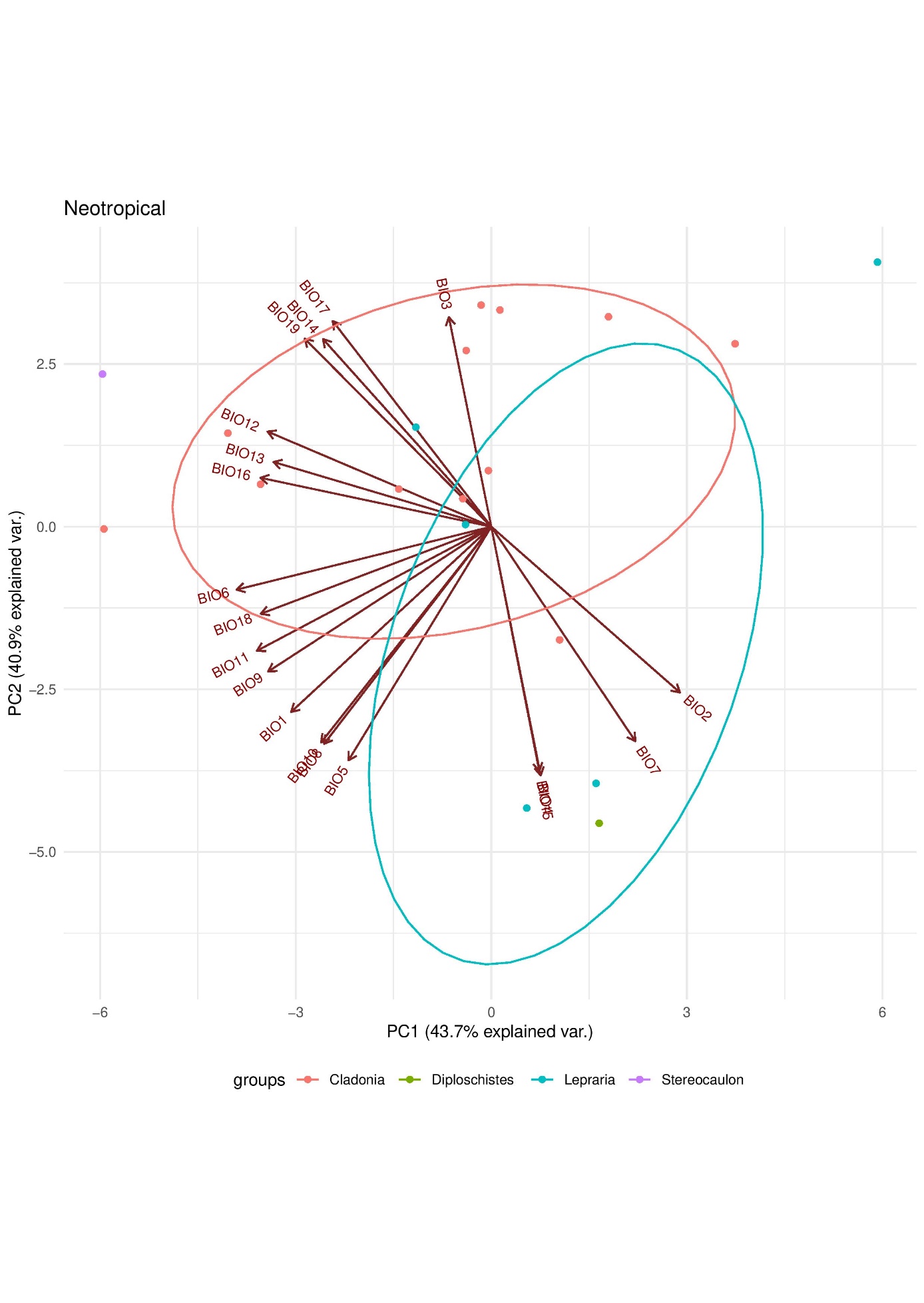


Figure S5. PCA result of *Asterochloris* distribution depending on climatic factors and taxonomic affiliation of lichen forming fungi for Pantropical lichens.


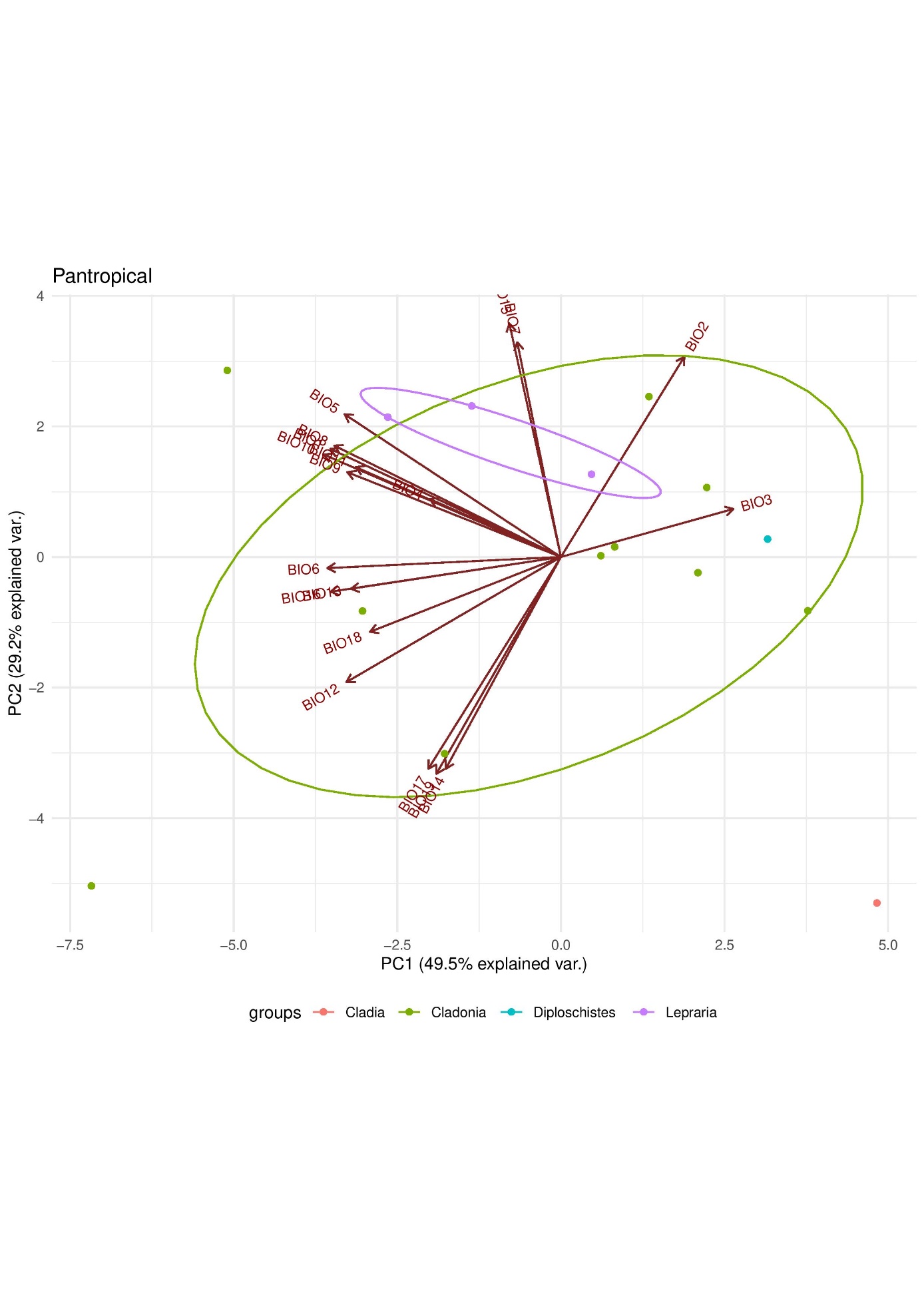


Figure S6. PCA result of *Asterochloris* distribution depending on climatic factors and habitat type; group 1 – lower montane cloud forest, group 2 – upper montane cloud forest section 1, group 3 – upper montane cloud forest section 2, group 4 – open high Andean vegetation.


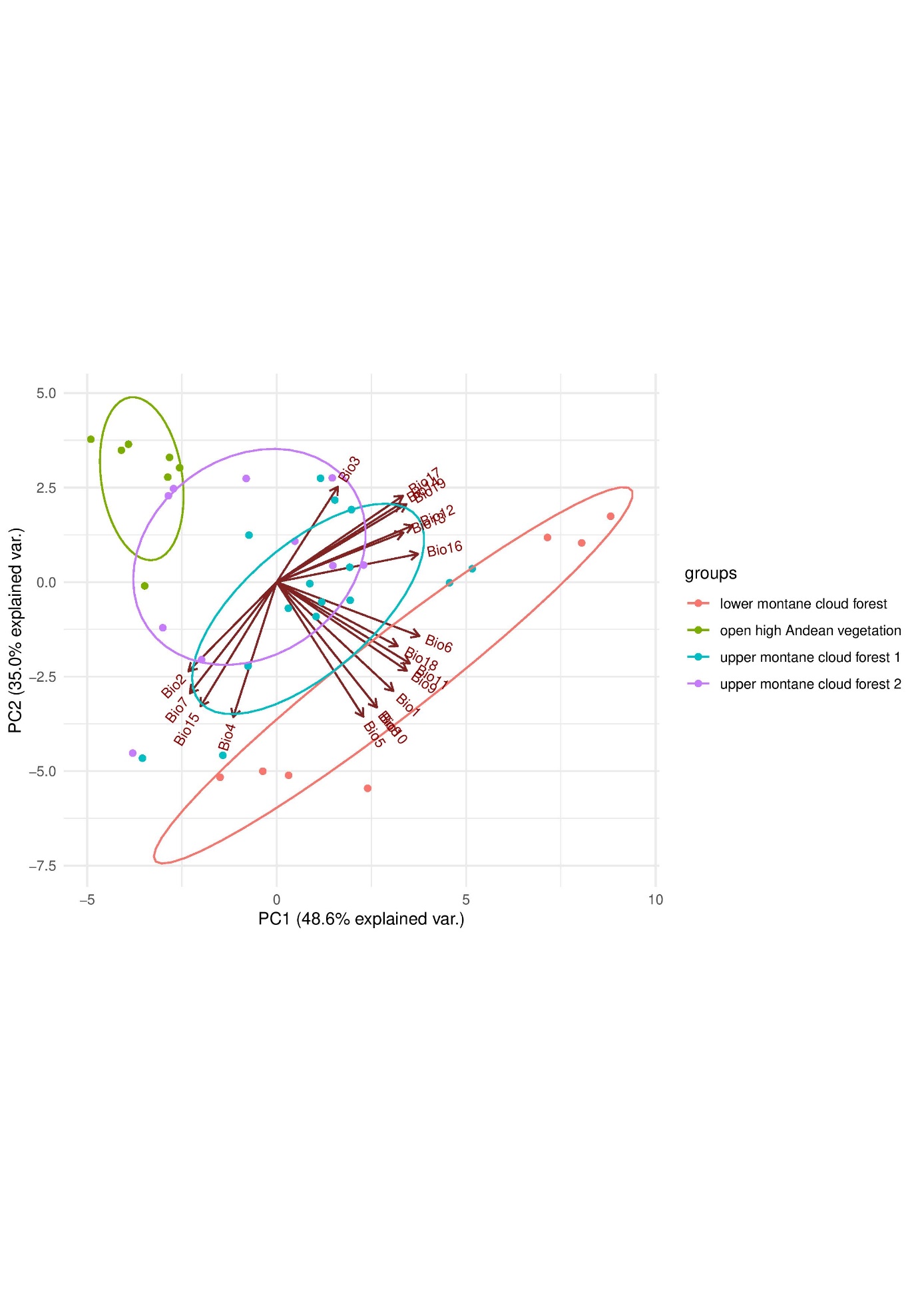


Figure S7. Box-plot diagram representing differences in climate of selected habitat types in Bolivia: A – lower montane cloud forest, B – upper montane cloud forest 1, C – upper montane cloud forest 2, C-open high Andean vegetation. Climatic data were obtained from the Global Climate Data – WorldClim. BIO1**=**Annual Mean Temperature (°C), BIO7= Temperature Annual Range (°C), BIO12= Annual Precipitation (mm), BIO17= Precipitation of Driest Quarter (mm).


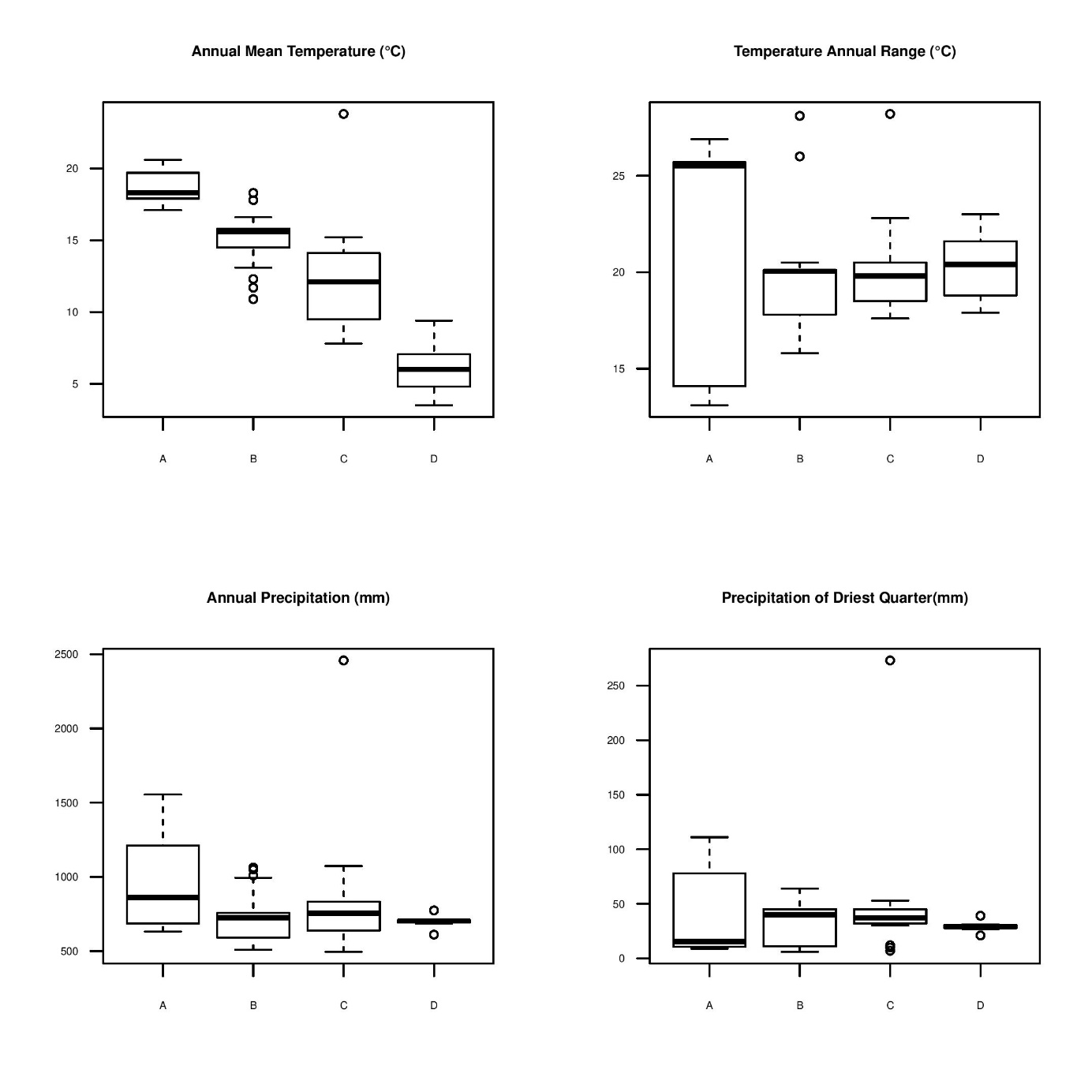


Table S1. List of specimens from which photobiont sequences were newly obtained with their ITS rDNA, *rbc*L and *actin* type I locus GenBank accession numbers. All samples were collected in Bolivia and voucher specimens are deposited in herbaria LPB and UGDA (Excel file, sheet 1.)

Table S2. Sequences of ITS rDNA, *rbc*L and *actin* type I gene of *Asterochloris* and *Vulcanochloris* downloaded from GenBank and used in this study in phylogenetic inference. Altitude above sea level, if not given in the publication, was determined based on a given geographic coordinates using the gps converter ([www.gps-coordinates.net](http://www.gps-coordinates.net)) (Excel file, sheet 2.). Sequences used for the analysis, the results of which are shown in Figure 1, are marked in gray. In yellow are marked the sequences from Bolivia, from research Pino-Bodas and Stenroos used in this study.

Table S3. The PCR condition used in this study to amplify the ITS rDNA region, the chloroplast *rbc*L gene, and the *actin* type I gene were amplified.

| Region | | ITS rDNA region | | chloroplast *rbc*L gene | | | *actin* type I gene |
| --- | --- | --- | --- | --- | --- | --- | --- |
| Primer name | | nr-SSU-1780-5’and ITS4 | a-nu-ssu-1752-5’and ITS4 | PRASF1 and  a-ch-rbcL-991-30-MPN | a-ch-rbcL-203-50-MPN and a-ch-rbcL-991-30-MPN | rbcLa and rbcLb | ActinF2 Astero-5’and ActinR2 Astero-3’ |
| initial denaturation | | 95°C for 2 min | 95°C for 2 min | 95°C for 2 min | 95°C for 2 min | 95°C for 2 min | 95°C for 2 min |
| 35 cycles | denaturation | 95°C for 1 min | 95°C for 1 min | 95°C for 1 min | 95°C for 1 min | 95°C for 1 min | 95°C for 1 min |
|  | annealing | 60°C for 1 min | 60°C for 1 min | 50 °C for 1 min | 60°C for 1 min | 60°C for 1 min | 60°C for 1 min |
|  | elongation | 72°C for 1 min | 72°C for 1 min | 72°C for 1 min | 72°C for 1 min | 72°C for 1 min | 72°C for 1 min |
| final extension | | 72°C for 7 min | 72°C for 7 min | 72°C for 7 min | 72°C for 7 min | 72°C for 7 min | 72°C for 7 min |

Table S4. Substitution models selected for each partition of *Asterochloris* and *Vulcanochloris* dataset using the Akaike Information Criterion (AIC) as implemented in PartitionFinder 2 (Lanfear et al., 2016).

| Partition names | organism | Partition length | Best Model |
| --- | --- | --- | --- |
| ITS1 rDNA | *Asterochloris* | 186 | GTR+I+G |
| 5.8S rDNA | *Asterochloris* | 123 | JC+I |
| ITS2 rDNA | *Asterochloris* | 203 | TVM+I+G |
| Intron1 of actin type I gene | *Asterochloris* | 247 | GTR+G |
| Exon part of actin type I gene | *Asterochloris* | 140 | TRNEF+G |
| Intron2 of actin type I gene | *Asterochloris* | 392 | GTR+G |
| rbcL | *Asterochloris* | 1325 | TVM+I+G |
| ITS1 rDNA | *Vulcanochloris* | 193 | SYM+G |
| 5.8S rDNA | *Vulcanochloris* | 169 | SYM+G |
| ITS2 rDNA | *Vulcanochloris* | 171 | SYM+G |

Table S5. Patterns of distribution of selected lichen forming fungi species and list of occurrences of examined photobionts. Occurrences with lack of precise GPS information are marked with grey.

| Mycobiont species | Mycobiont species range | Quotations for photobiont |
| --- | --- | --- |
| *Cladonia confusa* | Pantropical | Bolivia (this study, Pino-Bodas and Stenroos, 2020), Brazil (Cordeiro et al., 2005), France-Reunion, New Caledonia, New Zealand, South Africa (Pino-Bodas and Stenroos, 2020) |
| *Cladia aggregata* | Pantropical | Bolivia (this study), Chile (Piercey-Normore and Depriest,2001) |
| *Cladonia delavayi* | Pantropical | India (Ridka et al., 2014) |
| *Cladonia didyma* | Pantropical | Bolivia (this study), Brazil (Beilke et al.  unpublished), France-Reunion, Indonesia-Papua, Portugal-Azores (Pino-Bodas and Stenroos, 2020), USA: Alabama (Piercey-Normore and Depriest, 2001), USA: Hawaii (Pino-Bodas and Stenroos, 2020) |
| *Cladonia fruticulosa* | Pantropical | India (Ridka et al., 2014), New Zealand (Pino-Bodas and Stenroos, 2020) |
| *Cadonia melanopoda* | Pantropical | Bolivia (this study, Pino-Bodas and Stenroos, 2020) |
| *Cladonia praetermissa* | Pantropical | India (Ridka et al., 2014) |
| *Diploschistes hypoleucus* | Pantropical | Bolivia (this study) |
| *Lepraria pallida* | Pantropical | Bolivia (this study) |
| *Lepraria sipmaniana* | Pantropical | Bolivia (this study) |
| *Lepraria achariana* | Neotropical | Bolivia (this study) |
| *Cladonia* aff. *ahtii* | Neotropical | Bolivia (this study) |
| *Cladonia andesita* | Neotropical | Bolivia (this study) |
| *Cladonia arcuata* | Neotropical | Bolivia (this study), Costa Rica (Pino-Bodas and Stenroos, 2020) |
| *Cladonia calycantha* | Neotropical | Bolivia (this study, Pino-Bodas and Stenroos, 2020) |
| *Cladonia ceratophylla* | Neotropical | Bolivia (this study), France-Reunion (Pino-Bodas and Stenroos, 2020) |
| *Cladonia crinita* | Neotropical | Brazil (Cordeiro et al., 2005) |
| *Cladonia dactylota* | Neotropical | Bolivia (this study, Pino-Bodas and Stenroos, 2020), Costa Rica (Pino-Bodas and Stenroos, 2020) |
| *Cladonia fissidens* | Neotropical | Brazil (Cordeiro et al., 2005) |
| *Cladonia granulosa* | Neotropical | Bolivia (this study, Pino-Bodas and Stenroos, 2020) |
| *Cladonia isabellina* | Neotropical | Bolivia (this study, Pino-Bodas and Stenroos, 2020), Costa Rica (Pino-Bodas and Stenroos, 2020) |
| *Diploschistes* cf. *cinereocesius* | Neotropical | Bolivia (this study) |
| *Lepraria cryptovouauxii* | Neotropical | Bolivia (this study) |
| *Lepraria* aff. *hodkinsoniana* | Neotropical | Bolivia (this study) |
| *Lepraria impossibilis* | Neotropical | Bolivia (this study) |
| *Stereocaulon microcarpum* | Neotropical | Bolivia (this study) |
| *Stereocaulon pachycephalum* | Neotropical | Bolivia (this study) |
| *Cladonia arbuscula* | cosmopolitan | Czech Republic (Báckor et al., 2010), Bolivia (this study), USA: North Carolina Alabama (Piercey-Normore and Depriest, 2001), Slovakia (Škaloud & Peksa 2010). |
| *Cladonia chlorophaea* | cosmopolitan | Bolivia (this study), Canada (Beiggi & Piercey-Normore, 2007, Piercey-Normore and Depriest, 2001), Russia (Pino-Bodas and Stenroos, 2020) |
| *Cladonia coccifera* | cosmopolitan | Austria (Steinová et al., 2019), Czech Republic (Báckor et al., 2010, Škaloud et al., 2015, Steinová et al., 2019), Denmark (Steinová et al., 2019), France-Reunion (Pino-Bodas and Stenroos, 2020), Finland (Steinová et al., 2019), Norway (Steinová et al., 2019), Russia (Piercey-Normore and Depriest, 2001), Spain (Škaloud et al., 2015), UK (Steinová et al., 2019) |
| *Cladonia coniocraea* | cosmopolitan | Canada (Beiggi & Piercey-Normore, 2007), Nepal (Ridka et al., 2014), Czech Republic (Báckor et al., 2010) |
| *Cladonia furcata* | cosmopolitan | Czech Republic (Báckor et al., 2010), Bolivia (this study), India (Ridka et al., 2014), USA: Virginia (Piercey-Normore and Depriest, 2001) |
| *Cladonia pocillum* | cosmopolitan | Bolivia (Pino-Bodas and Stenroos, 2020), Canada (Beiggi & Piercey-Normore, 2007), Norway: Svalbard (Zang et al., 2015) |
| *Cladonia pyxidata* | cosmopolitan | Antarctica (Kim et al., 2017), Canada (Beiggi & Piercey-Normore, 2007), Czech Republic (Báckor et al., 2010), India (Ridka et al., 2014), Italy (Piercey-Normore and Depriest, 2001), New Zealand (Pino-Bodas and Stenroos, 2020), |
| *Lepraria congesta* | cosmopolitan | Bolivia (this study) |
| *Lepraria finkii* | cosmopolitan | Bolivia (this study), USA: Wisconsin (Nelsen & Gargas, 2006, USA (Nelsen & Gargas, 2008) |
| *Stereocaulon alpinum* | cosmopolitan | Argentina (Vancurova et al., 2018), Austria (Vancurova et al., 2018), Bolivia (this study), Canada (Vancurova et al., 2018), Denmark (Vancurova et al., 2018), Finland (Vancurova et al., 2018), Georgia (Vancurova et al., 2018), Greenland, Iceland (Vancurova et al., 2018) |
| *Stereocaulon myriocarpum* | cosmopolitan | Bolivia (this study), Costa Rica (Vancurova et al., 2018), Venezuela (Vancurova et al., 2018) |
| *Stereocaulon tomentosum* | cosmopolitan | Bolivia (this study), USA: Alaska (Vancurova et al., 2018), Slovakia (Báckor et al., 2010) |

Table S6. Results of distance-based redundancy analyzes (dbRDA) used to select statistically significant predicators for explaining variation for each data sets used in variation partitioning analyzes.

| Variable/Data | | Bolivian species (N=77) | Stereocaulon (N=169) | Cladonia (N=179) | Lepraria (N=35) | Cosmopolitan  species (N=75) | Neotropical species (N=23) | Pantropical species (N=20) |
| --- | --- | --- | --- | --- | --- | --- | --- | --- |
| Altitude | | 0.005 ** | 0.005 ** | 0.005 ** | Pr>F | 0.005** | Pr>F | Pr>F |
| Habitat | | 0.005 ** | - | - | - | - | - | - |
| Substrate | | Pr>F | - | - | - | 0.005** | Pr>F | Pr>F |
| Geographical distance | PCNM1 | 0.010 ** | 0.005 ** | 0.005 ** | 0.005 ** | 0.005 ** | Pr>F | 0.005 ** |
|  | PCNM2 | 0.005 ** | 0.005 ** | 0.005 ** | Pr>F | 0.005 ** | 0.005 ** | Pr>F |
|  | PCNM3 | 0.010 ** | 0.005 ** | 0.005 ** | Pr>F | Pr>F | Pr>F | Pr>F |
|  | PCNM4 | Pr>F | 0.005 ** | 0.005 ** | Pr>F | 0.010 ** | Pr>F | 0.005 ** |
|  | PCNM5 | 0.020 * | 0.015 * | 0.005 ** | Pr>F | 0.005 ** | Pr>F | 0.030 * |
|  | PCNM6 | Pr>F | 0.005 ** | 0.040 * | Pr>F | Pr>F | Pr>F | Pr>F |
|  | PCNM7 | Pr>F | 0.005 ** | 0.005 ** | Pr>F | 0.035 * | Pr>F | Pr>F |
|  | PCNM8 | 0.025 * | 0.005 ** | 0.030 * | Pr>F | Pr>F | Pr>F | Pr>F |
|  | PCNM9 | 0.005 ** | Pr>F | 0.025 * | Pr>F | 0.035 * | Pr>F | Pr>F |
|  | PCNM10 | Pr>F | Pr>F | 0.045 * | Pr>F | Pr>F | Pr>F | Pr>F |
| Climate | BIO1 | Pr>F | 0.005 ** | 0.005 ** | Pr>F | 0.005 ** | Pr>F | Pr>F |
|  | BIO2 | Pr>F | Pr>F | 0.005 ** | 0.005 ** | 0.010 ** | Pr>F | Pr>F |
|  | BIO3 | 0.015 * | 0.005 ** | 0.005 ** | Pr>F | 0.005 ** | Pr>F | Pr>F |
|  | BIO4 | 0.005 ** | Pr>F | 0.010 ** | Pr>F | 0.005 ** | 0.005 ** | 0.005 ** |
|  | BIO5 | Pr>F | Pr>F | 0.005 ** | Pr>F | Pr>F | Pr>F | Pr>F |
|  | BIO6 | Pr>F | 0.005 ** | 0.005 ** | Pr>F | Pr>F | Pr>F | Pr>F |
|  | BIO7 | Pr>F | 0.005 ** | Pr>F | Pr>F | Pr>F | Pr>F | Pr>F |
|  | BIO8 | Pr>F | 0.005 ** | 0.005 ** | Pr>F | 0.005 ** | Pr>F | Pr>F |
|  | BIO9 | 0.005 ** | 0.005 ** | Pr>F | Pr>F | 0.005 ** | Pr>F | Pr>F |
|  | BIO10 | Pr>F | 0.005 ** | 0.005 ** | 0.005 ** | 0.040 * | Pr>F | Pr>F |
|  | BIO11 | Pr>F | Pr>F | 0.010 ** | Pr>F | 0.010 ** | Pr>F | Pr>F |
|  | BIO12 | Pr>F | Pr>F | 0.005 ** | Pr>F | Pr>F | Pr>F | Pr>F |
|  | BIO13 | 0.050* | Pr>F | Pr>F | Pr>F | Pr>F | Pr>F | 0.005 ** |
|  | BIO14 | Pr>F | 0.005 ** | 0.005 ** | Pr>F | Pr>F | Pr>F | Pr>F |
|  | BIO15 | Pr>F | 0.010 ** | 0.005 ** | Pr>F | Pr>F | Pr>F | Pr>F |
|  | BIO16 | Pr>F | 0.040 * | Pr>F | Pr>F | Pr>F | Pr>F | Pr>F |
|  | BIO17 | Pr>F | 0.015 * | Pr>F | Pr>F | 0.005 ** | Pr>F | Pr>F |
|  | BIO18 | Pr>F | Pr>F | Pr>F | Pr>F | Pr>F | Pr>F | Pr>F |
|  | BIO19 | 0.045 * | 0.005 ** | Pr>F | Pr>F | Pr>F | Pr>F | Pr>F |

Table S7. Results of variation partitioning analyses for Bolivian data and selected genera of mycobionts, i.e. *Stereocaulon*, *Cladonia* and *Lepraria.* Values > 0.1 are in bold.

| Variable/Data | Bolivian species-habitat (N=54) | Bolivian species-altitude (N=54) | *Stereocaulon* (N=169) | *Cladonia* (N=179) | *Lepraira* (N=35) |
| --- | --- | --- | --- | --- | --- |
| Species of mycobiont | **0.25** | **0.26** | **0.12** | **0.31** | **0.35** |
| Climate | 0.06 | 0.06 | 0.00 | 0.05 | 0.05 |
| Altitude/Habitat | 0.00 | 0.00 | 0.08 | 0.00 | - |
| Geographical distance | 0.03 | 0.03 | 0.04 | 0.03 | 0.05 |
| Species of mycobiont + Climate | 0.00 | 0.00 | 0.01 | 0.00 | 0.00 |
| Species of mycobiont + Altitude/Habitat | 0.02 | 0.03 | 0.01 | 0.00 | - |
| Species of mycobiont + Geographical distance | 0.02 | 0.00 | 0.06 | 0.01 | 0.00 |
| Climate + Altitude/Habitat | 0.00 | 0.00 | 0.00 | 0.00 | - |
| Climate + Geographical distance | 0.00 | 0.00 | 0.00 | 0.02 | 0.00 |
| Altitude/Habitat + Geographical distance | 0.00 | 0.00 | 0.02 | 0.00 | - |
| Species of mycobiont + Climate + Altitude/Habitat | 0.05 | 0.05 | 0.00 | **0.11** | - |
| Species of mycobiont + Climate + Geographical distance | 0.03 | 0.03 | 0.00 | 0.02 | 0.06 |
| Species of mycobiont + Altitude/Habitat + Geographical distance | 0.01 | 0.00 | 0.01 | 0.02 | - |
| Climate + Altitude/Habitat + Geographical distance | 0.00 | 0.00 | 0.03 | 0.00 | - |
| Species of mycobiont + Climate + Altitude/Habitat + Geographical distance | 0.00 | 0.00 | 0.01 | 0.01 | - |
| Residuals | **0.53** | **0.54** | **0.61** | **0.42** | **0.49** |

Table S8. Results of variation partitioning analyses for selected group of lichen forming fungi representing different distribution pattern: cosmopolitan, Neotropical, Pantropical. Values > 0.1 are in bold.

| Variable/Data | | Cosmopolitan  species (N=77) | Neotropical species (N=30) | Pantropical species (N=23) |
| --- | --- | --- | --- | --- |
| Mycobiont+ Geographical distance | Mycobiont | **0.07** | **0.59** | **0.59** |
|  | Geographical distance | 0.07 | 0.00 | 0.00 |
|  | Mycobiont + Geographical distance | 0.00 | **0.17** | **0.21** |
|  | Residual | **0.86** | **0.24** | **0.20** |
| Mycobiont+ Altitude | Mycobiont | **0.13** | - | - |
|  | Altitude | 0.01 | - | - |
|  | Mycobiont + Altitude | 0.00 | - | - |
|  | Residual | **0.86** | - | - |
| Mycobiont+ Substrate | Mycobiont | **0.13** | - | - |
|  | Substrate | 0.01 | - | - |
|  | Mycobiont + Substrate | 0.00 | - | - |
|  | Residual | **0.86** | - | - |
| Mycobiont+ Climate | Mycobiont | 0.00 | **0.59** | **0.59** |
|  | Climate | **0.15** | 0.00 | 0.00 |
|  | Mycobiont + Climate | 0.00 | **0.20** | **0.30** |
|  | Residual | **0.85** | **0.21** | **0.11** |

Table S9. List of records of *Asterochloris* species or lineages. Records lacking precise GPS information are marked in grey.

| Species or lineage of *Asterochloris* | Country | Species of mycobiont |
| --- | --- | --- |
| *Asterochloris mediterranea* | Bolivia,  Cape Verde, France-Reunion Germany,  Greece, Iran, Italy, Portugal, Azores,  Russia, Spain, Sweden, Turkey, Yugoslavia | ***Cladonia calycantha* (this study)**  *Cladonia cervicornis* s. str. (Moya et al., 2015)  *Cladonia convoluta/foliacea* (Moya et al., 2015, Pino-Bodas et al. unpublished)  *Cladonia rangiformis* (Moya et al., 2015, Piercey-Normore and DePriest, 2001, Pino-Bodas and Stenroos, 2020)  *Ramalina farina*cea (Moya et al., 2017)  ***Stereocaulon pachycephalum* (this study)**  *Stereocaulon vesuvianum* (Vančurová et al. 2018)  *Cladonia fimbriata* (Piercey-Normore and DePriest, 2001)  *Cladonia symphycarpa* (Wedin et al., 2015)  *Diploschistes muscorum* (Wedin et al., 2015) *Lepraria nylanderiana* (Nelsen and Gargas, 2008)  *Cladonia corsicana* (Pino-Bodas and Stenroos, 2020)  *Cladonia rei* (Pino-Bodas and Stenroos, 2020)  *Cladonia glauca* (Pino-Bodas and Stenroos, 2020)  *Cladonia cryptochlorophaea* (Pino-Bodas and Stenroos, 2020)  *Cladonia crispata* (Pino-Bodas and Stenroos, 2020)  *Cladonia diversa* (Pino-Bodas and Stenroos, 2020)  *Cladonia foliacea* (Pino-Bodas and Stenroos, 2020)  *Cladonia ramulosa s. lat.* (Pino-Bodas and Stenroos, 2020)  *Cladonia confusa* (Pino-Bodas and Stenroos, 2020)  *Cladonia didyma* (Pino-Bodas and Stenroos, 2020) |
| *Asterochloris friedlii* | Bolivia, Canada, China, Czech Republic, Finland, Korea, Romania, Slovakia, USA | *Lepraria caesioalba* (Škaloud and Peksa, 2010, Nelsen and Gargas, 2008)  *Lepraria* cf. *caesioalba* (Škaloud and Peksa, 2010)  *Lepraria cupressicola* (Nelsen and Gargas, 2008)  ***Lepraria finkii*** **(this study**, Nelsen and Gargas, 2006*,* 2008)  ***Lepraria achariana*** **(this study)**  *Cladonia caespiticia* (Nelsen and Gargas, 2006)  *Lepraria caesiella* (Nelsen and Gargas, 2008) *Lepraria elobata* (Škaloud et al., 2015)  *Lepraria incana* (Nelsen and Gargas, 2008)  *Lepraria rigidula* (Peksa and Škaloud, 2011)  *Lepraria nivalis* (Nelsen and Gargas, 2008)  *Myelochroa aurulenta* (MH258948)  *Hypotrachyna minarum* (KM250371)  *Cladonia fimbriata* (Pino-Bodas and Stenroos, 2020) |
| *Asterochloris* sp. clade 12 | Bolivia, Canada, Czech Republic, India, Mexico, Russia, USA: Alaska | ***Cladonia chlorophaea* (this study)**  *Cladonia fimbriata* (Báckor et al., 2010)  *Cladonia furcata* (Báckor et al., 2010)  *Cladonia pyxidata* (Báckor et al., 2010, Řídká et al., 2014)  *Cladonia rei* (Báckor et al., 2010)  *Cladonia subulata* (Báckor et al., 2010)  *Diploschistes muscorum* (Báckor et al., 2010)  *Stereocaulon dactylophyllum* (Vančurová et al. 2018)  *Stereocaulon paschale* (Nelsen and Gargas, 2006)  *Stereocaulon rivulorum* (Vančurová et al. 2018)  *Cladonia cornuta* (Beiggi and Piercey-Normore, 2007)  *Cladonia pocillum* (Beiggi and Piercey-Normore, 2007, Pino-Bodas and Stenroos, 2020)  *Cladonia bacilliformis* (Pino-Bodas and Stenroos, 2020) |
| *Asterochloris* sp. clade 9 | Bolivia, Brazil, Cameroon, Costa Rica, France-Reunion,  Guyana,  India, Madagascar, Nepal, New Zealand, Portugal-Azores,  Scotland, USA: Florida, Georgia, North Carolina, South Carolina | ***Cladonia arcuata* (this study)**  *Cladonia cervicornis* subsp. *verticillata* (Cordeiro et al., 2005, Řídká et al., 2014)  *Cladonia confusa* (Beilke et al. unpublished, Cordeiro et al., 2005, Guzow-Krzemińska and Stocker-Wörgötter, 2013)  *Cladonia coniocraea* (Řídká et al., 2014)  ***Cladonia dactylota*** (**this study,** Pino-Bodas and Stenroos, 2020)  *Cladonia delavayi* (Řídká et al., 2014)  *Cladonia evansii* (Yahr et al., 2004)  *Cladonia fruticulosa* (Řídká et al., 2014)  ***Cladonia granulosa*** (**this study,** Pino-Bodas and Stenroos, 2020)  *Cladonia leporina* (Yahr et al., 2004)  *Cladonia praetermissa* (Řídká et al., 2014)  *Cladonia scabriuscula* (Řídká et al., 2014)  *Cladonia spinea* (Piercey-Normore and DePriest, 2001)  *Cladonia subtenuis* (Yahr et al., 2004, 2006)  *Cladia aggregata* (Nelsen and Gargas, 2008)  *Cladonia crispatula* (Beilke et al. unpublished)  *Cladonia didyma* (Beilke et al. unpublished, Pino-Bodas and Stenroos, 2020)  *Cladonia rappii* (Piercey-Normore and DePriest, 2001)  *Cladonia variegata* (Piercey-Normore and DePriest, 2001)  *Cladonia subradiata* (Pino-Bodas and Stenroos, 2020)  *Cladonia polycarpia* (Pino-Bodas and Stenroos, 2020)  *Cladonia hypoxantha* (Pino-Bodas and Stenroos, 2020)  *Cladonia ravenelii* (Pino-Bodas and Stenroos, 2020)  *Cladonia compressa* (Pino-Bodas and Stenroos, 2020)  *Cladonia pityrophylla* (Pino-Bodas and Stenroos, 2020)  *Cladonia candelabrum* (Pino-Bodas and Stenroos, 2020)  *Cladonia strepsilis* (Pino-Bodas and Stenroos, 2020)  *Cladonia mauritiana* (Pino-Bodas and Stenroos, 2020)  *Cladonia subreticulata* (Pino-Bodas and Stenroos, 2020)  *Cladonia ceratophyllina* (Pino-Bodas and Stenroos, 2020)  *Cladonia medusina* (Pino-Bodas and Stenroos, 2020)  *Cladonia coccifera* (Pino-Bodas and Stenroos, 2020) |
| *Asterochloris* sp. P2 | Bolivia, Costa Rica | ***Cladia aggregata* (this study)**  ***Cladonia arbuscula* ubsp. *boliviana* (this study)**  ***Cladonia calycantha* (this study)**  ***Cladonia didyma* (this study)**  ***Cladonia furcata* (this study)**  ***Cadonia melanopoda* (this study)**  ***Cladonia* cf. *subradiosa* (this study)**  *Pilophorus* cf. *cereolus* (Nelsen and Gargas, 2006)  ***Stereocaulon myriocarpum*** **(this study)**  *Cladonia kriegeri* (Pino-Bodas and Stenroos, 2020)  *Cladonia subsquamosa* (Pino-Bodas and Stenroos, 2020)  *Cladonia mexicana* (Pino-Bodas and Stenroos, 2020) |
| *Asterochloris* sp. clade StA1 | Bolivia, Costa Rica, Denmark | ***Cladonia andesita* (this study)**  ***Cladonia confusa* (this study)**  ***Cladonia isabellina*** (**this study,** Pino-Bodas and Stenroos, 2020)  *Cladonia melanopoda* (Pino-Bodas and Stenroos, 2020)  *Cladonia cartilaginea* (Pino-Bodas and Stenroos, 2020)  *Cladonia leprocephala* (Pino-Bodas and Stenroos, 2020)  *Stereocaulon rivulorum* (Vančurová et al. 2018)  *Stereocaulon vesuvianum* (Vančurová et al. 2018) |
| *Asterochloris* sp. clade StA5 | Austria, Bolivia, Canada,  Georgia | ***Cladonia pocillum*** **(this study)**  *Stereocaulon alpinum* (Vančurová et al. 2018)  *Stereocaulon* cf. *grande* (Vančurová et al. 2018)  *Stereocaulon vesuvianum* (Vančurová et al. 2018) |
| *Asterochloris* sp. clade A14 | Bolivia, Panama | ***Cladonia* cf. *ramulosa* (this study)**  ***Lepraria* cf. *vouauxii* (this study**)  *Stereocaulon* cf. *obesum* (Vančurová et al. 2018) |
| *Asterochloris* sp. clade L54 | Costa Rica, Bolivia | ***Lepraria hodkinsoniana*** **(this study)**  *Lepraria* sp. (Nelsen and Gargas, 2008) |
| *Asterochloris* sp. clade A6 | Bolivia, Costa Rica, Czech Republic | *Lepraria crassissima* (Peksa and Škaloud, 2011)  ***Lepraria cryptovouauxii*** **(this study)**  *Lepraria nigrocincta* (Nelsen and Gargas, 2008) |
| *Asterochloris* sp. clade S1 | Bolivia,  Costa Rica | ***Cladonia didyma*** **(this study)**  *Cladonia microscypha* (Pino-Bodas and Stenroos, 2020)  *Cladonia arcuata* (Pino-Bodas and Stenroos, 2020)  *Cladonia cortilaginea s. lat.* (Pino-Bodas and Stenroos, 2020)  *Cladonia andesita* (Pino-Bodas and Stenroos, 2020)  *Cladonia dactylota* (Pino-Bodas and Stenroos, 2020)  *Cladonia meridensis* (Pino-Bodas and Stenroos, 2020)  *Cladonia diversa* (Pino-Bodas and Stenroos, 2020)  *Stereocaulon* sp. (Nelsen and Gargas, 2006)  ***Stereocaulon tomentosum* (this study)** |

Table S10. List of haplotypes showing relationships between ITS rDNA sequences of *Asterochloris* associating with *Stereocaulon* spp. Sequences from tropical region are marked in green.

| Haplotype 1 | **AF345382** *A. glomerata* from *S. evolutoides*, USA: Massachusetts |
| --- | --- |
| Haplotype 2 | **AF345404**, **AF345405** *A. glomerata* from *S. pileatum*, USA: Massachusetts,  **FJ626732** *A. glomerata* from *S. evolutoides*, USA: Massachusetts,  **FM945392** *A. glomerata* from *S. saxatile*, Slovakia,  **MH415204** *A. glomerata* from *S. pileatum*, Slovakia,  **KP314550** *A. glomerata* from *S. vesuvianum*, Svalbard,  **MH415301** *A. glomerata* from *S. arcticum*, Iceland,  **MH415302** *A. glomerata* from *S*. cf. *alpinum*, South Argentina,  **MH415393** *A. glomerata* from *S*. cf. *grande*, Finland,  **MH415396** *A. glomerata* from *S. symphycheilum*, Czech Republic,  **MH415433** *A. glomerata* from *S. rivulorum*, DenmarkAF345405 |
| Haplotype 3 | **AF345442** *A. erici* from *S. dactylophyllum*, Canada |
| Haplotype 4 | **AM905993** *A. excentrica* from *S. dactylophyllum*, USA: Vermont |
| Haplotype 5 | **DQ229880**, **MH415326** *A. irregularis* from *S. botryosum*, USA: Alaska,  **DQ229881** *A. irregularis* from *S. subcoralloides*, USA: Alaska,  **KP314615** *A. irregularis* from *S. botryosum*, Svalbard,  **MH415205** *A. irregularis* from *S. vesuvianum*, Slovakia,  **MH415259**, **MH415262**, **MH415265**, **MH415266**, **MH415270**, **MH415271** *A. irregularis*  from *S. arcticum*, Iceland  **MH415260**, **MH415261**, **MH415267**, **MH415300** *A. irregularis* from *S. vesuvianum*, Iceland,  **MH415268** *A. irregularis* from *S*. cf. *alpinum*, Iceland,  **MH415272** *A. irregularis* from *S. vesuvianum*, Japan,  **MH415310**, **MH415387**, **MH415413** *A. irregularis* from *S. vesuvianum*, Czech Republic,  **MH415314**, **MH415321** *A. irregularis* from *S. apocalypticum*, USA: Alaska,  **MH415315**, **MH415316**, **MH415317** A. irregularis from S. intermedium, USA: Alaska,  **MH415318**, **MH415319**, **MH415320**, **MH415325**, **MH415332**, **MH415335**  *A. irregularis* from *S. paschale*, USA: Alaska,  **MH415356**, **MH415362** *A. irregularis* from *S. paschale*, Greenland,  **MH415358**, **MH415360**, **MH415361** *A. irregularis* from *S. alpinum*, Greenland,  **MH415359** *A. irregularis* from *S*. cf. *arcticum*, Greenland,  **MH415368** *A. irregularis* from *S. tomentosum/sasakii*, USA: Alaska,  **MH415377** *A. irregularis* from *S. rivulorum*, Denmark,  **MH415392** *A. irregularis* from *S*. cf. *paschale*, Finland,  **MH415394** *A. irregularis* from *S. alpinum*, Finland,  **MH415397**, **MH415398** *A. irregularis* from *S. symphycheilum*, Czech Republic,  **MH415399** *A. irregularis* from *S. paschale*, Russia,  **MH415405** *A. irregularis* from *S. vesuvianum*, Norway,  **MH415406** *A. irregularis* from *S. dactylophyllum*, Finland,  **MH415306**, **MH415308**, **MH415309**, **MH415340** A. irregularis from S. sp., Russia  **MH415324**, **MH415327**, **MH415328**, **MH415331**, **MH415333**, **MH415336**  *A. irregularis* from *S*. sp., USA: Alaska,  **MH415363** *A. irregularis* from *S*. sp., Greenland,  **AF345411** *A. irregularis* from *S*. sp., Iceland |
| Haplotype 6 | **DQ229882** *A*. sp. clade StA4 from *S. paschale*, Canada,  **MH415357**, *A*. sp. clade StA4 from *S. alpinum*, Greenland,  **MH415281** *A*. sp. clade StA4 from *S*. sp., Sweden |
| Haplotype 7 | **DQ229885** *A*. *pseudoirregularis* from *S. vesuvianum*, USA: Alaska,  **MH415323** *A*. *pseudoirregularis* from *S. tomentosum/sasakii*, USA: Alaska |
| Haplotype 8 | **DQ229886** *A*. sp clade S3 from *S. saxatile*, USA: Alaska |
| Haplotype 9 | **DQ229887** *A*. sp. clade 12 from *S. paschal*e, USA: Alaska,  **MH415322** *A*. sp. clade 12 from *S. rivulorum*, USA: Alaska |
| Haplotype 10 | **KJ690281** *A*. sp. clade 12 from *S. paschal*e, Canada |
| Haplotype 11 | **KJ690289** *A*. sp. clade 12 from *S. paschal*e, Canada |
| Haplotype 12 | **KP314536** *A*. *pseudoirregularis* from *S. vesuvianum*, Svalbard,  **KP314583** *A*. *pseudoirregularis* from *S. botryosum*, Svalbard,  **MH415263** *A*. *pseudoirregularis* from *S. arcticum*, Iceland,  **MH415367** *A*. *pseudoirregularis* from *S. paschal*e, USA: Alaska,  **MH415378** *A*. *pseudoirregularis* from *S. arcticum*, Denmark,  **MH415407** *A*. *pseudoirregularis* from *S. pileatum*, Austria,  **MH415408** *A*. *pseudoirregularis* from *S*. cf. *alpinum*, Austria,  **MH415409** *A*. *pseudoirregularis* from *S. alpinum*, Canada |
| Haplotype 13 | **KP314586** *A*. *pseudoirregularis* from *S. botryosum*, Svalbard |
| Haplotype 14 | **KP318667** *A. woessiae* from *S*. cf. *azoreum*, Spain, Canary Island,  **MH415232**, **MH415235**, **MH415418** *A. wossieae* from *S. azoreum*, Portugal, Mareida,  **MH415234**, **MH415237**, **MH415244** A*. woessiae* from *Stereocaulon* sp., Portugal, Mareida,  **MH415383** *A. woessiae* from *S. alpinum*, Denmark,  **MH415379** *A. woessiae* from *S*. sp., Denmark |
| Haplotype 15 | **MH415203** *A*. *stereocaulonicola* from *S. nanodes*, Slovakia |
| Haplotype 16 | **MH415206** *A. lobophora* from *S. nanodes*, Slovakia,  **MH415297**, **MH415298**, **MH415299** *A. lobophora* from *S. alpinum*, Georgia,  **FM945391** *A. lobophora* from *Stereocaulon tomentosum*, Slovakia |
| Haplotype 17 | **MH415210** *A*. sp. T20 from *S. vesuvianum*, Tanzania |
| Haplotype 18 | **MH415212** *A*. sp. clade StA3 from *S. vesuvianum*, Tanzania |
| Haplotype 19 | **MH415216**, **MH415273** *A*. sp. clade StA6 from *S*. cf. *alpinum*, South Argentina |
| Haplotype 20 | **MH415217** *A. italiana* from *S. alpinum*, South Argentina |
| Haplotype 21 | **MH415218** *A*. *stereocaulonicola* from *S*. cf. *vesuvianum*, South Argentina,  **MH415290** *A*. *stereocaulonicola* from *S. vesuvianum*, South Argentina,  **MT036569**, **MT036570**, **MT036571** *A. stereocaulonicola* from *S. alpinum*, Antarctica |
| Haplotype 22 | **MH415219** *A*. sp. clade StA7 from *S*. cf. *vesuvianum*, South Argentina |
| Haplotype 23 | **MH415221**, **MH415222**, **MH415224**, **MH415225** *A*. sp clade StA5 from *S. alpinum*, Georgia,  **MH415282** *A*. sp clade StA5 from *S. vesuvianum*, Georgia,  **MH415291** *A*. sp clade StA5 from *S*. cf. *alpinum*, Georgia,  **MH415366** *A*. sp clade StA5 from *S. alpinum*, Austria |
| Haplotype 24 | **MH415223** *A*. *stereocaulonicola* from *S*. cf. *alpinum*, South Argentina,  **MH415226** *A*. *stereocaulonicola* from *S. vesuvianum*, South Argentina,  **MH415274** *A*. *stereocaulonicola* from *S. vesuvianum*, Italy,  **MH415303** *A*. *stereocaulonicola* from *S*. sp., South Argentina |
| Haplotype 25 | **MH415229** *A*. sp. clade StA8 from *S. myriocarpum*, Costa Rica |
| Haplotype 26 | **MH415230**, **MH415231**, **MH415246**, **MH415253**, **MH415254**, **MH415423**  *A. italiana* from *S. azoreum*, Portugal, Madeira,  **MH415339** *A. italiana* from *S. condensatum*, Germany,  **MH415439** *A. italiana* from *S. rivulorum*, Denmark |
| Haplotype 27 | **MH415238**, **MH415424** *A. wossieae* from *S. azoreum*, Portugal, Mareida, |
| Haplotype 28 | **MH415252** *A. wossieae* from *S. azoreum*, Portugal, Mareida,  **MH415419**, **MH415425**, **MH415426** *A. wossieae* from *S.* *pileatum*, Portugal, Mareida |
| Haplotype 29 | **MH415255** *A*. aff. *italiana* from *S*. sp., Portugal, Madeira,  **MH415422** *A*. aff. *italiana* from *S. azoreum*, Portugal, Madeira,  **MH415391** *A*. aff. *italiana* from *S. dactylophyllum*, Sweden |
| Haplotype 30 | **MH415257**, **MH415258** *A*. *antarctica* from *S. alpinum*, Iceland |
| Haplotype 31 | **MH415264** *A*. *stereocaulonicola* from *S*. cf. *alpinum*, Iceland |
| Haplotype 32 | **MH415269** *A*. *antarctica* from *S. saxatile*, Iceland |
| Haplotype 33 | **MH415287** *A*. sp. clade StA8 from S*. myriocarpum*, Venezuela |
| Haplotype 34 | **MH415288** *A*. sp. clade StA8 from *S. myriocarpum*, Venezuela |
| Haplotype 35 | **MH415294** *A*. sp. clade StA8 from S*. myriocarpum*, Venezuela |
| Haplotype 36 | **MH415296** *A*. sp. clade A14 from *S*. cf. *obesum*, Panama |
| Haplotype 37 | **MH415313** *A. mediterranea* from *S. vesuvianum*, Spain, Canary Island |
| Haplotype 38 | **MH415334** *A. phycobiontica* from *S. alpinum*, Austria |
| Haplotype 39 | **MH415364** *A*. sp. clade StA4 from *S. alpinum*, Greenland,  **MH415329**, **MH415330** *A*. sp. clade StA4 from *S*. sp., USA: Alaska |
| Haplotype 40 | **MH415369** *A. irregulari*s from *S. symphycheilum*, Austria,  **MH415370** *A. irregularis* from *S. vesuvianum*, Austria,  **MH415434** *A. irregularis* from *S. vesuvianum*, Denmark,  **MH415279** *A. irregularis* from *S*. sp., Austria |
| Haplotype 41 | **MH415375**, **MH415382**, **MH415386**, **MH415390**, **MH415430**, **MH415431**, **MH415437**  *A*. sp. clade StA1 from *S. rivulorum*, Denmark,  **MH415376**, **MH415384**, **MH415385**, **MH415428**,  **MH415429**, **MH415432**, **MH415436** *A*. sp. clade StA1 from *S. vesuvianum*, Denmark,  **MH415381** *A*. sp. clade StA1 from *S*. cf. *vesuvianum*, Denmark,  **MH415388**, **MH415389**, **MH415435** *A*. sp. clade StA1 from *S*. cf. *rivulorum*, Denmark,  **MH415380** *A*. sp. clade StA1 from *S*. sp., Denmark |
| Haplotype 42 | **MH415395** *A*. sp. clade 12 from *S. dactylophyllum*, CzechRepublic |
| Haplotype 43 | **MH415410** *A*. sp. StA5 from *S. alpinum*, Canada,  **MH415411**, **MH415412** *A*. sp. clade StA5 from *S*. cf. *grande*, Canada,  **MH415414** *A*. sp. clade StA5 from *S*. cf. *alpinum*, Canada |
| Haplotype 44 | **MH415415** *A*. sp. clade 8 from *S*. cf. *grande*, Canada,  **MH415451** *A*. sp. clade 8 from *S. dactylophyllum*, Czech Republic |
| Haplotype 45 | **MH415427** *A. glomerata* from *S. pileatum*, Portugal, Madeira |
| Haplotype 46 | **Kukwa 14827** *A*. sp. clade Bol 7 from *S. alpinum*, La Paz 1 |
| Haplotype 47 | **UGDA-L 18555** *A*. sp. clade Bol 4 from *S. microcarpum*, La Paz 3 |
| Haplotype 48 | **UGDA-L 18613** *A*. sp. clade P2 from *S. myriocarpum*, La Paz 4 |
| Haplotype 49 | **UGDA-L 18566** *A. mediterranea* from *S. pachycephalum*, La Paz 4 |
| Haplotype 50 | **UGDA-L 25173** *A*. sp. clade S1 from *S. tomentosum*, Santa Cruz 1 |
| Haplotype 51 | **MH415220** *A*. sp. from *S*. sp., Argentina |
| Haplotype 52 | **MH415307** *A. lobophora* from *S*. sp., Russia |
| Haplotype 53 | **MH415374** *A*. sp. clade 12 from *S*. sp., Mexico |
| Haplotype 54 | **DQ229884** *A*. sp. clade S1 from *S*. sp., Costa Rica |
| Haplotype 55 | **AF345433** *A. excentrica* from *S. dactylophyllum*, USA: Vermont |

Table S11. List of haplotypes showing relationships between ITS rDNA sequences of *Asterochloris* associating with *Cladonia* spp. Sequences from tropical region are marked in green.

| Haplotype number | Sample ID |
| --- | --- |
| Haplotype 1 | **Kukwa 14684** *A*. sp. clade StA1 from *C. andesita*, La Paz 1 |
| Haplotype 2 | **Kukwa** **16168**, **Kukwa 16909** *A*. sp. clade MN082 form *C*. aff. *ahtii*, Chuquisaca 2,  **Kukwa** **14785**, *A*. sp. clade MN082 from *C. ceratophylla*, La Paz 1,  **Kukwa** **18470** *A*. sp. clade MN082 from *C. ceratophylla*, Santa Cruz 1 |
| Haplotype 3 | **Kukwa** **14886** *A*. sp. clade P2 from *C. calycantha*, La Paz 2, |
| Haplotype 4 | **Kukwa** **14890** *A*. sp. clade A14 from *C*. cf. *ramulosa*, La Paz 2 |
| Haplotype 5 | **HE803050** *A*. sp. clade 12 from *C. pyxidata*, India  **Kukwa** **15040** *A*. sp. clade 12 from *C. chlorophaea*, Cochabamba 1 |
| Haplotype 6 | **Kukwa** **15044** *A*. sp. clade P2 from *C. furcata*, Cochabamba 1 |
| Haplotype 7 | **UGDA-L** **19015** *A*. sp. clade 9 from *C. dactylota*, Santa Cruz 1,  **Kukwa** **16205** *A*. sp. clade 9 from *C. granulosa*, Chuquisaca 2 |
| Haplotype 8 | **Kukwa** **16553** *A*. sp. clade Bol 5 from *C. furcata*, Tarija 1 |
| Haplotype 9 | **Kukwa** **16621** *A*. sp. clade StA1 from *C. confusa*, Tarija 1 |
| Haplotype 10 | **UGDA-L** **18961** *A. mediterranea* from *C. calycantha*, La Paz 3 |
| Haplotype 11 | **UGDA-L** **18963** *A*. sp. clade StA5 from *C. pocillum*, La Paz 5,  **AF345427** *A*. sp. clade StA5 from *C. subulata*, Germany |
| Haplotype 12 | **UGDA-L** **18987** *A*. sp. clade P2 from *C. melanopoda*, La Paz 4,  **UGDA-L** **19021** *A*. sp. clade P2 from *C. arbuscula* subsp. *boliviana*, Santa Cruz 1,  **Kukwa** **14880a** *A*. sp. clade P2 from *C.* cf. *subcariosa*, La Paz 2,  **UGDA-L** **19004** *A*. sp. clade P2 from *C. didyma*, La Paz 4 |
| Haplotype 13 | **Kukwa 15030b** *A*. sp. clade StA1 from *C. confusa*, Cochabamba 1,  **UGDA-L** **18992** *A*. sp. clade StA1 from *C. isabellina*, La Paz 4 |
| Haplotype 14 | **UGDA-L** **18999** *A*. sp. clade S1 from *C. didyma*, La Paz 2 |
| Haplotype 15 | **UGDA-L** **19009** *A*. sp. clade 9 from *C. arcuata*, Santa Cruz 2 |
| Haplotype 16 | **AF345375** *A. glomerata* from *C. strepsilis*, Canada,  **AF345391** *A. glomerata* from *C. stellaris*, USA: North Carolina,  **AF345392** *A. glomerata* from *C. atlantica*, USA: Massachusetts  **AF345393** *A. glomerata* from *C. atlantica*, USA: Alabama,  **AF345394** *A. glomerata* from *C. cervicornis* subsp. *verticillata*, USA: North Carolina,  **AF345395** *A. glomerata* from *C. caroliniana*, USA: Alabama,  **AF345397** *A. glomerata* from *C. grayi*, Canada,  **AF345399** *A. glomerata* from *C. strepsilis*, Japan,  **AF345398**, **AF345400** *A. glomerata* from *C. strepsilis*, USA: North Carolina,  **AY622822**, **AY622826**, **AY622831**, **AY622832**, **AY622833** *A. glomerata* from  *C. gracilis*, Canada  **AY622836**, **AY622837**, **AY622838**, **AY622839**, **AY622840** *A. glomerata* from  *C. rangiferina*, Canada,  **AY712688** *A. glomerata* from *C. subsetacea*, USA: Florida,  **DQ530178** *A. glomerata* from *C. cervicornis* subsp. *verticillata*, Canada  **DQ530181** *A. glomerata* from *C. pyxidata*, Canada,  **FM945351**, **FM945352**, **FM945353** *A. glomerata* from *C. coccifera*, Czech Republic,  **FM945357** *A. glomerata* from *C. deformis*, Czech Republic,  **FM945363**, **FM945364** *A. glomerata* from *C. macilenta*, Czech Republic,  **FM945367**, **FM945368** *A. glomerata* from *C. mitis*, Czech Republic,  **FM945372** *A. glomerata* from *C*. cf. *novochlorophaea*, Czech Republic,  **KP031547** *A. glomerata* from *C. rangiferina*,  **FM945370**, **FM945371**, **KT989951**, **KT989953**, **KT989955**, **KT989956**, **KT989957**, **KT989958**, **KT989959**, **KT989960**, **KT989961**, **KT989964**, **KT989965** *A. glomerata* from *C. pleurota*, Czech Republic,  **KT989952** *A. glomerata* from *C. pleurota*, Austria,  **KT989954** *A. glomerata* from *C. pleurota*, Romania,  **KT989962** *A. glomerata* from *C. pleurota*, Germany,  **KT989963** *A. glomerata* from *C. pleurota*, Finland,  **KT989966** *A. glomerata* from *C. pleurota*, Denmark |
| Haplotype 17 | **AF345376**, **AF345379**, **AF345380** *A. glomerata* from *C. grayi*, Canada,  **AF345377** *A. glomerata* from *C. botrytes*, Canada,  **AF345378** *A. glomerata* from *C. crispata*, Canada,  **AF345381** *A. glomerata* from *C. squamosa*, Canada,  **AY622824** *A glomerata* from *C. gracilis*, Canada |
| Haplotype 18 | **AF345383** *A. glomerata* from *C. strepsilis*, Italy  **AY712689** *A. glomerata* from *C. dimorphoclada*, USA: Florida,  **DQ530182** *A. glomerata* from *C. ochrochlora*, Canada,  **DQ530186** *A. glomerata* from *C. merochlorophaea*, Canada,  **HE803028** *A. glomerata* from *C. coniocraea*, Nepal,  **KT989941**, **KT989945**, **KT989949** *A. glomerata* from *C. pleurota*, Austria,  **KT989942**, **KT989943**, **KT989944**, **KT989950**, **KT989967** *A. glomerata* from  *C. pleurota*, Czech Republic  **KT989946**, **KT989947** *A. glomerata* from *C. deformis*, Finland,  **KT989948** *A. glomerata* from *C. deformis*, Czech Republic |
| Haplotype 19 | **AF345384** *A. glomerata* from *C. arbuscula*, USA: North Carolina |
| Haplotype 20 | **AF345385** *A. glomerata* from *C. grayi*, Canada |
| Haplotype 21 | **AF345386** *A. glomerata* from *C. robbinsii*, USA: North Carolina |
| Haplotype 22 | **AF345387** *A. glomerata* from *C. strepsilis*, USA: North Carolina |
| Haplotype 23 | **AF345388** *A. glomerata* from *C. strepsilis*, Canada,  **DQ530187** *A. glomerata* from *C. grayi*, Canada |
| Haplotype 24 | **AF345390** *A. glomerata* from *C. arbuscula*, USA: North Carolina |
| Haplotype 25 | **AF345396** *A. glomerata* from *C. floridana*, USA: Massachusetts |
| Haplotype 26 | **AF345401**, **AF345402** *A. glomerata* from *C. strepsilis*, Canada |
| Haplotype 27 | **AF345408** *A. irregulari*s from *C. cenotea*, Russia,  **AF345409** *A. irregularis* from *C. coccifera*, Russia,  **AF345410** *A. irregularis* from *C. stellaris*, Finland,  **AM906000** *A. irregularis* from *C. arbuscula*, Slovakia,  **AY622821**, **AY622823**, **AY622827**, **AY622828**, **AY622829** *A. irregularis* from  *C. gracilis*, Canada,  **FM945347** *A. irregularis* from *C. arbuscula*, Czech Republic,  **KT989923**, **KT989924**, **KT989935** *A. irregularis* from *C. pleurota*, Czech Republic,  **KT989925**, **KT989926** *A. irregulari*s from *C. pleurota*, Finland,  **KT989927** *A. irregularis* from *C. diversa*, Belgium,  **KT989928** *A. irregularis* from *C. deformis*, Finland,  **KT989929**, **KT989930** *A. irregularis* from *C. coccifera*, Finland,  **KT989931**, **KT989934** *A. irregularis* from *C. pleurota*, Norway,  **KT989932** *A. irregularis* from *C. deformis*, Czech Republic,  **KT989933** *A. irregularis* from *C. coccifera*, Czech Republic |
| Haplotype 28 | **AF345412** *A*. sp. IO49 from *C. uncialis,* Finland,  **AY622830** *A*. sp. MN274 from *C. gracilis*, Canada,  **AY622835** *A*. sp. MN611 from *C. rangiferina*, Canada |
| Haplotype 29 | **AF345413** *A*. *pseudoirregularis* from *C. bellidiflora*, Norway,  **AY622825** *A*. *pseudoirregularis* from *C. gracilis*, Canada,  **AY622834** *A*. *pseudoirregularis* from *C. rangiferina*, Canada,  **FM945376** *A*. *pseudoirregularis* from *C. rangiferina*, Czech Republic  **KP314531**, **KP314538**, **KP314541**, **KP314544**, **KP314584**, **KP314600**, **KP314659**, **KP314664**  *A*. *pseudoirregularis* from *C. borealis*, Svalbard,  **KP314585**, **KP314593**, **KP314649**, **KP314657** *A*. *pseudoirregularis* from *C. mitis*, Svalbard,  **KT989936** *A*. *pseudoirregularis* from *C. coccifera*, Norway,  **KT989937** *A*. *pseudoirregularis* from *C. pleurota*, Montenegro,  **KT989938** *A*. *pseudoirregularis* from *C. pleurota*, Austria,  **KT989939** *A*. *pseudoirregularis* from *C. pleurota*, Norway |
| Haplotype 30 | **AF345414** *A*. sp. IO50 from *C. gracilis*, Russia |
| Haplotype 31 | **AF345415** *A*. sp. clade MN082 from *C. didyma*, USA: Alabama |
| Haplotype 32 | **AF345416** *A*. sp. clade A14 from *C. peltastica*, French Guyana, |
| Haplotype 33 | **AF345417** *A*. sp. clade 9 from *C. rappii*, USA: North Carolina,  **AY712696** *A*. sp. clade 9 from *C. evansii*, USA: Florida |
| Haplotype 34 | **AF345418** *A*. sp. clade IH2 from *C. spinea*, French Guyana,  **AF345419** *A*. sp. clade IH2 from *C. variegata*, French Guyana,  **HE803043** *A*. sp. clade IH2 from *C. scabriuscula*, India |
| Haplotype 35 | **AF345420** *A. italiana* from *C. glauca*, Denmark,  **AF345421** *A. italiana* from *C. capitellata*, Austria,  **AF345422** *A. italiana* from *C. staufferi*, Austria,  **KT989890** *A. italiana* from *C. coccifera*, Czech Republic,  **KT989889**, **KT989899** *A. italian*a from *C. diversa*, Czech Republic,  **KT989891**, **KT989900**, **KT989901**, **KT989902** *A. italiana* from *C. diversa*, Denmark,  **KT989892** *A. italiana* from *C. diversa*, Belgium,  **KT989893** *A. italiana* from *C. diversa*, Netherlands,  **KT989894**, **KT989903** *A. italiana* from *C. diversa*, Spain,  **KT989895**, **KT989904** *A. italiana* from *C. diversa*, Norway,  **KT989896**, **KT989897** *A. italiana* from *C. diversa*, UK,  **KT989898** *A. italiana* from *C. diversa*, Austria |
| Haplotype 36 | **AF345424** *A. italiana* from *C. scabriuscula*, Austria, |
| Haplotype 37 | **AF345425** *A*. *lobophora* from *C. cristatella*, USA: Alabama |
| Haplotype 38 | **AF345426** *A*. *lobophora* from *C. parasitica*, Canada,  **AY712703**, **AY712706** *A*. *lobophora* from *C. pachycladodes*, USA: Florida,  **DQ530179** *A. lobophora* from *C. coniocraea*, Canada,  **DQ530184** *A. lobophora* from *C. pocillum*, Canada,  **DQ530189** *A. lobophora* from *C. fimbriata*, Canada,  **FM945354** *A. lobophora* from *C. coniocraea*, Czech Republic  **FM945356**, **FM945387** *A. lobophora* from *C. rei*, Czech Republic,  **FM945366** *A. lobophora* from *C. macilenta*, Czech Republic,  **FM945373** *A. lobophora* from *C. pyxidata*, Czech Republic,  **FM945377** *A. lobophora* from *C. rangiformis*, Czech Republic,  **FM945379** *A. lobophora* from *C. subulata*, Czech Republic,  **KP318674**, **KT989907** *A. lobophora* from *C. coccifera*, Czech Republic,  **KT989906** *A. lobophora* from *C. diversa*, Czech Republic |
| Haplotype 39 | **AF345428** *A*. sp. clade 8 from *C. turgida*, Russia |
| Haplotype 40 | **AF345429** *A*. sp. clade 8 from *C. furcata*, USA: Virginia |
| Haplotype 41 | **AF345430** *A*. sp. clade 8 from *C. symphycarpa*, Finland, |
| Haplotype 42 | **AF345431**, **DQ530188** *A*. sp. clade 8 from *C. chlorophaea,* Canada,  **FM945358** *A*. sp. clade 8 from *C. fimbriata*, Slovakia,  **FM945365** *A*. sp. clade 8 from *C. macilenta*, Czech Republic,  **FM945383** *A*. sp. clade 8 from *C. subulata*, Czech Republic,  **FM945386** *A*. sp. clade 8 from *C. rei*, Czech Republic,  **KT989916** *A*. sp. clade 8 from *C. coccifera*, Austria,  **KT989917**, **KT989918** *A*. sp. clade 8 from *C. coccifera*, Czech Republic |
| Haplotype 43 | **AF345432** *A*. sp. clade 8 from *C. farinacea*, USA: New York |
| Haplotype 44 | **AF345434** *A. mediterrane*a from *C. fimbriata*, Sweden |
| Haplotype 45 | **AF345435** *A. mediterranea* from *C. rangiformis*, Yugoslavia, |
| Haplotype 46 | **AF345436** *A*. sp. clade 12 from *C. pyxidata*, Italy |
| Haplotype 47 | **AF345438** *A. italiana* from *C. ochrochlora*, Australia |
| Haplotype 48 | **AF345439**, **AF345440**, **AF345441** *A. erici* from *C. cristatella*, USA: Massachusetts |
| Haplotype 49 | **AF345443** *A*. sp. MN068 from *C. pulviniformis*, French Guyana,  **JN990658,** *A*. sp. from *C. didyma*, Brasil |
| Haplotype 50 | **AM906016** *A. woessiae* from *C. foliacea*, Czech Republic,  **FM945348**, **FM945350** *A. woessiae* from *C. humilis*, Czech Republic,  **FM945385** *A. woessiae* from *C. subulata*, Czech Republic,  **KP318669** *A. woessiae* from *C. coccifera*, Spain |
| Haplotype 51 | **AY712690** *A*. sp. clade 9 from *C. subtenuis*, USA: Florida |
| Haplotype 52 | **AY712691** *A*. sp. clade 9 from *C. evansii*, USA: Florida |
| Haplotype 53 | **AY712692** *A*. sp. clade 9 from *C. evansii*, USA: Florida |
| Haplotype 54 | **AY712693** *A*. sp. clade 9 from *C. evansii*, USA: Florida |
| Haplotype 55 | **AY712694** *A*. sp. clade 9 from *C. subtenuis*, USA: Florida |
| Haplotype 56 | **AY712695** *A*. sp. clade 9 from *C. leporina*, USA: Florida |
| Haplotype 57 | **AY712697** *A*. sp. clade 9 from *C. subtenuis*, USA: Florida |
| Haplotype 58 | **AY712698** *A*. sp. clade 9 from *C. subtenuis*, USA: Florida |
| Haplotype 59 | **AY712699** *A*. sp. clade 9 from *C. subtenuis*, USA: Florida |
| Haplotype 60 | **AY712700** *A*. sp. clade 9 from *C. subtenuis*, USA: Florida |
| Haplotype 61 | **AY712701** *A*. *lobophora* from *C. perforata*, USA: Florida |
| Haplotype 62 | **AY712702** *A*. *lobophora* from *C. prostrata*, USA: Florida |
| Haplotype 63 | **AY712704** *A*. *lobophora* from *C. pachycladodes*, USA: Florida |
| Haplotype 64 | **AY712705** *A*. *lobophora* from *C. prostrata*, USA: Florida |
| Haplotype 65 | **AY712707**, **AY712710** *A. lobophora* from *C. pachycladodes*, USA: Florida,  **AY842280** *A. lobophora* from *C. perforata*, USA: Florida, |
| Haplotype 66 | **AY712708** *A. lobophora* from *C. leporina*, USA: Florida |
| Haplotype 67 | **AY712709** *A. lobophora* from *C. prostrata*, USA: Florida |
| Haplotype 68 | **AY712711** *A*. *lobophora* from *C. pachycladodes*, USA: Florida |
| Haplotype 69 | **AY842276** *A*. sp. clade 9 from *C. verticillaris*, Brasil |
| Haplotype 70 | **AY842277** *A*. sp. from *C. crinita*, Brazil |
| Haplotype 71 | **AY842278** *A*. sp. from *C. fissidens*, Brasil |
| Haplotype 72 | **AY842279** *A*. sp. clade 9 from *C. confusa*, Brasil,  **JQ359767** *A*. sp. clade 9 from *C. confusa* |
| Haplotype 73 | **DQ229879** *A. friedlii* from *C. caespitecia*, USA: Pennsylvania |
| Haplotype 74 | **DQ482671** *A. lobophora* from *C. subtenuis*, USA: Missouri |
| Haplotype 75 | **DQ482672** *A*. sp. RY1242 from *C. subtenuis*, USA: Virginia |
| Haplotype 76 | **DQ482673** *A*. sp. RY1240 from *C. subtenuis*, USA: Virginia |
| Haplotype 77 | **DQ482674** *A*. *lobophora* from *C. subtenuis*, USA: Arkansas |
| Haplotype 78 | **DQ482675** *A*. *lobophora* from *C. subtenui*s, USA: North Carolina |
| Haplotype 79 | **DQ482676** *A*. sp. RY1225 from *C. subtenuis*, USA: Pennsylvania |
| Haplotype 80 | **DQ482677** *A*. sp. clade 8 from *C. subtenuis*, USA: Missouri |
| Haplotype 81 | **DQ482678** *A*. sp. clade 8 from *C. subtenuis*, USA: North Carolina, |
| Haplotype 82 | **DQ482679** *A*. sp. clade 8 from *C. subtenuis*, USA: Virginia |
| Haplotype 83 | **DQ482680** *A*. sp. clade 8 from *C. subtenuis*, USA: Pennsylvania |
| Haplotype 84 | **DQ482681** *A*. sp. clade 9 from *C. subtenuis*, USA: Georgia |
| Haplotype 85 | **DQ482682** *A*. sp. clade 9 from *C. subtenuis*, USA: Florida |
| Haplotype 86 | **DQ530180** *A*. sp. clade 12 from *C. cornuta*, Canada  **DQ530190** *A*. sp. clade 12 from *C. pocillum*, Canada  **FM945355**, **FM945378**, **FM945381** *A*. sp. clade 12 from *C. rei*, Czech Republic  **FM945359** *A*. sp. clade 12 from *C. fimbriata*, Czech Republic  **FM945360**, **FM945361**, **FM945362** *A*. sp. clade 12 from *C. furcata*, Czech Republic,  **FM945374**, **FM945375** *A*. sp. clade 12 from *C. pyxidata*, Czech Republic  **FM945384** *A*. sp. clade 12 from *C. subulata*, Czech Republic |
| Haplotype 87 | **DQ530183** *A. glomerata* from *C. ecmocyna*, Canada |
| Haplotype 88 | **DQ530185** *A. lobophora* from *C. macrophyllodes*, Canada |
| Haplotype 89 | **DQ530191** *A. leprarii* from *C. pocillum*, Canada |
| Haplotype 90 | **FM205720** *A. mediterranea* from *C. convoluta/foliacea*, Portugal,  **KP257384**, **KP257385**, **KP257386**, **KP257388**, **KP257389**, **KP257390**, **KP257391**, **KP257392** *A. mediterrane*a from *C. convoluta/foliacea*, Spain,  **KP257395** *A. mediterranea* from *C. rangiformis*, Spain,  **KP257396** *A. mediterranea* from *C. cervicornis*, Spain,  **KT215300**, **KT215301**, **KT215302**, **KT215309**, **KT215310**, **KT215311** *A. mediterranea* from *C. symphycarpa*, Sweden,  **KT215306**, **KT215307** *A. mediterranea* from *C. symphycarpa*, Germany |
| Haplotype 91 | **FM945349** *A*. aff. *italiana* from *C. humilis*, Czech Republic,  **KT989908**, **KT989909**, **KT989910**, **KT989914** *A*. aff. *italiana* from *C. coccifera*, Czech Republic,  **KT989911** *A*. aff. *italiana* from *C. coccifera*, Austria,  **KT989912** *A*. aff. *italiana* from *C. diversa*, Germany,  **KT989913** *A*. aff. *italiana* from *C. diversa*, Spain |
| Haplotype 92 | **FM945369** *A. lobophora* from *C. ochrochlora*, Czech Republic,  **FM945382** *A. lobophora* from *C. rei*, Czech Republic |
| Haplotype 93 | **FM945380** *A*. sp. clade 8 from *C. rei*, |
| Haplotype 94 | **HE803029** *A*. sp. clade I1 from *C. rangiferina*, India,  **HE803030** *A*. sp. clade I1 from *C. furcata*, India |
| Haplotype 95 | **HE803032** *A*. sp. clade I1 from *C. furcata*, India,  **HE803031** *A*. sp. clade I1 from *C. pyxidata*, India,  **HE803033**, **HE803034** *A*. sp. clade I1 from *C. corymbescens*, India |
| Haplotype 96 | **HE803035**, **HE803036** *A*. sp. clade I2 from *C. furcata*, India |
| Haplotype 97 | **HE803037** *A*. sp. clade I2 from *C. furcata*, India |
| Haplotype 98 | **HE803038** *A*. sp. clade 9 from *C. scabriuscula*, India |
| Haplotype 99 | **HE803039** *A*. sp. clade 9 from *C. coniocraea*, Nepal,  **HE803040** *A*. sp. clade 9 from *C. delavayi*, India |
| Haplotype 100 | **HE803041** *A*. sp. clade 9 from *C. verticillata*, India, |
| Haplotype 101 | **HE803042** *A*. sp. clade 9 from *C. delavayi*, India |
| Haplotype 102 | **HE803044** *A*. sp. clade 9 from *C. verticillata*, India  **JN990660** *A*. sp. clade 9 from *C. didyma*, Brasil |
| Haplotype 103 | **HE803045**, **HE803046**, **HE803047** *A*. sp. clade 9 from *C. fruticulosa*, India |
| Haplotype 104 | **HE803048** *A*. sp. clade 9 from *C. praetermiss*a, India |
| Haplotype 105 | **HE803049** *A*. sp. clade 9 from *C. cariosa*, India |
| Haplotype 106 | **JN990651** *A*. sp. clade 9 from *C. confusa*, Brasil |
| Haplotype 107 | **JN990652** *A*. sp. clade 9 from *C. confusa*, Brasil,  **JN990657** *A*. sp. clade 9 from *C. crispatula*, Brasil |
| Haplotype 108 | **JN990653** *A*. sp. clade 9 from *C. confusa*, Brasil |
| Haplotype 109 | **JN990654** *A*. sp. clade 9 from *C. crispatula*, Brasil |
| Haplotype 110 | **JN990655** *A*. sp. clade 9 from *C. crispatula*, Brasil |
| Haplotype 111 | **JN990656** *A*. sp. clade 9 from *C. crispatula*, Brasil |
| Haplotype 112 | **JN990659** *A*. sp. clade 9 from *C. didyma*, Brasil |
| Haplotype 113 | **KJ690277** *A*. *irregularis* from *C. pleurota*, Canada |
| Haplotype 114 | **KJ690285** *A*. *irregularis* from *C. pleurota*, Canada |
| Haplotype 115 | **KP257366** *A. mediterranea* from *C. convoluta/foliacea*, Spain |
| Haplotype 116 | **KP257367**, **KP257368**, **KP257369**, **KP257371**, **KP257372**, **KP257373**, **KP257374**, **KP257375**, **KP257376**, **KP257377**, **KP257378**, **KP257380** *A. mediterrane*a from  *C. convoluta/foliacea*, Spain |
| Haplotype 117 | **KP257370** *A. mediterrane*a from *C. convoluta/foliacea*, Spain |
| Haplotype 118 | **KP257379**, **KP257387** *A. mediterranea* from *C. convoluta/foliacea*, Spain |
| Haplotype 119 | **KP257381** *A. mediterranea* from *C. convoluta/foliacea*, Spain,  **KP257393**, **KP257394** *A. mediterranea* from *C. rangiformis*, Spain,  **KP257397**, **KP257398** *A. mediterranea* from *C. cervicornis*, Spain |
| Haplotype 120 | **KP257382** *A. mediterranea* from *C. convoluta/foliacea*, Spain |
| Haplotype 121 | **KP257383** *A. mediterranea* from *C. convoluta/foliacea*, Spain |
| Haplotype 122 | **KP314492** *A. erici* from *C. pocillum*, Svalbard |
| Haplotype 123 | **KP314498**, **KP314501**, **KP314581**, **KP314595**, **KP314648** *A*. *magna* from *C. pocillum*, Svalbard |
| Haplotype 124 | **KP314503** *A. erici* from *C. pocillum*, Svalbard |
| Haplotype 125 | **KP314523** *A*. *magna* from *C. pocillum,* Svalbard |
| Haplotype 126 | **KP314558** *A*. *magna* from *C. pocillum*, Svalbard |
| Haplotype 127 | **KP314578** *A*. *pseudoirregularis* from *C. borealis*, Svalbard |
| Haplotype 128 | **KP314582** *A*. *magna* from *C. pocillum*, Svalbard |
| Haplotype 129 | **KP314590** *A*. *pseudoirregularis* from *C. borealis*, Svalbard |
| Haplotype 130 | **KP314598** *A. erici* from *C. pocillum*, Svalbard |
| Haplotype 131 | **KP314604** *A*. *magna* from *C. pocillum*, Svalbard |
| Haplotype 132 | **KP314633** *A. erici* from *C. pocillum*, Svalbard |
| Haplotype 133 | **KP314635** *A*. *magna* from *C. pocillum*, Svalbard |
| Haplotype 134 | **KP314665** *A*. *magna* from *C. pocillum*, Svalbard |
| Haplotype 135 | **KP318671** *A. echinata* from *C. diversa*, Portugal |
| Haplotype 136 | **KT215308** *A. mediterranea* from *C. symphycarpa*, Germany |
| Haplotype 137 | **KT989888** *A. italiana* from *C. coccifera*, Czech Republic |
| Haplotype 138 | **KT989905** *A*. sp. clade StA4 from *C.* *coccifera*, Czech Republic |
| Haplotype 139 | **KT989915** *A*. *antarctica* from *C. coccifera*, Austria  **MT036576** *A. antarctica* from *C*. sp., Antarctica |
| Haplotype 140 | **KT989919**, **KT989921** *A*. sp. clade StA3 from *C. coccifera*, Austria  **KT989920** *A*. sp. clade StA3 from *C. coccifera*, Czech Republic  **MH415438** *A*. sp. clade StA3 from *C. macrophylla*, Denmark |
| Haplotype 141 | **KT989922** *A*. *stereocaulonicola* from *C. coccifera*, Czech Republic |
| Haplotype 142 | **KT989940** *A*. *pseudoirregularis* from *C. deformis*, Spain |
| Haplotype 143 | **KX051235**, **KX051236**, **KX051238** *A. sejongensis* from *C. pyxidata*, Antarctica |
| Haplotype 144 | **MT036564**, **MT036566**, **MT036567**, **MT036568** *A. pseudoirregularis* from  *C*. sp., Antarctica,  **MT036565** *A. pseudoirregularis* from *C*. *gracilis*., Antarctica |
| Haplotype 145 | **MT036573**, **MT036574**, **MT036575** *A. antarctica* from *C*. sp., Antarctica |
| Haplotype 146 | **MW043596** *A*. sp. clade 9 from *C. hypoxantha*, USA: South Carolina  **MW043536** *A*. sp. clade 9 from *C. polycarpia,* USA: Georgia |
| Haplotype 147 | **MW043577** *A*. sp. clade 9 from *C. hypoxantha*, USA: South Carolina |
| Haplotype 148 | **MW043504** *A*. sp. clade 8 from *C. conista,* USA, Pensilvania  **MW043490** *A*. sp. clade 8 from *C. conista,* Finand |
| Haplotype 149 | **MW043528** *A. glomerata* from *C. brevis,* USA, Pensilvania  **MW043521** *A. glomerata* from *C. hondoensis*, Japan  **MW043633** *A. glomerata* from *C. pleurota*, Russia  **MW043653** *A. glomerata* from *C. subulata*, Finland  **MW043664** *A. glomerata* from *C. oricola*, Canada |
| Haplotype 150 | **MW043639** *A. glomerata* from *C. piedmontensis*, USA, Pensilvania  **MW043587** *A. glomerata* from *C. conspicua s. lat.,* USA: North Carolina  **MW043650** *A. glomerata* from *C. mitis*, Netherlands  **MW043638** *A. glomerata* from *C. monomorpha*, Finland  **MW043609** *A. glomerata* from *C. deformis*, Finland  **MW043603** *A. glomerata* from *C. uncialis subsp. uncialis*, Canada |
| Haplotype 151 | **MW043535** *A. glomerata* from *C. piedmontensis*, USA, Pensilvania |
| Haplotype 152 | **MW043537** *A. italiana* from *C. prolifica*, USA, Oregon  **MW043607** *A. italiana* from *C. islandica*, USA, Alaska  **MW043539** *A. italiana* from *C. pulvinata*, Netherlands  **MW043704** *A. italiana* from *C. portentosa*, Scotland  **MW043636** *A. italiana* from C. sp., New Zealand |
| Haplotype 153 | **MW043523** *A. italiana* from *C. poroscypha*, USA, Oregon |
| Haplotype 154 | **MW043589** *A*. sp. clade 9 from *C. petrophila,* USA: North Carolina |
| Haplotype 155 | **MW043588** *A. glomerata* from *C. caroliniana,* USA: North Carolina  **MW043586** *A. glomerata* from *C. submitis*, USA, New York |
| Haplotype 156 | **MW043697** *A*. sp. clade 8 from *C. apodocarpa*, USA, New York |
| Haplotype 157 | **MW043698** *A*. sp. clade 9 from *C. grayi*, USA, New York |
| Haplotype 158 | **MW043696** *A*. *glomerata* from *C. floerkeana,* USA, New York |
| Haplotype 159 | **MW043500** *A*. sp. clade 8 from *C. conista*, USA, New Jersey |
| Haplotype 160 | **MW043540** *A*. sp. clade 8 from *C. santensis,* USA, New Jersey |
| Haplotype 161 | **MW043509** *A. lobophora* from *C. rei*, USA, Minnesota |
| Haplotype 162 | **MW043563** *A*. sp. clade 9 from *C. skottsbergii*, USA, Hawaii |
| Haplotype 163 | **MW043562** *A*. sp. clade MN082 from *C. didyma*, USA, Hawaii  **MW043691**, **MW043694** *A*. sp. clade MN082 from *C. nana*, Portugal, Azores  **MW043687** *A*. sp. clade MN082 from *C. subradiata*, Portugal, Azores |
| Haplotype 164 | **MW043576** *A. lobophora* from *C. leporina*, USA, Georgia |
| Haplotype 165 | **MW043573** *A. lobophora* from *C. leporina*, USA, Georgia |
| Haplotype 166 | **MW043575** *A*. sp. clade 9 from *C. ravenelii*, USA, Florida |
| Haplotype 167 | **MW043549** *A*. sp. clade 9 from *C. varians*, France, Reunion |
| Haplotype 168 | **MW043497** *A*. sp. clade 8 from *C. conista*, USA, Connecticut |
| Haplotype 169 | **MW043703**, **MW043701** *A. italiana* from *C. portentosa*, USA, Alaska  **MW043612** *A. italiana* from *C. groenlandica*, USA, Alaska  **MW043702** *A. italiana* from *C. portentosa*, Scotland  **MW043706** *A. italiana* from *C. polydactyla*, Scotland  **MW043681**, **MW043672** *A. italiana* from *C. rigida*, New Zealand  **MW043675**, **MW043674**, **MW043673**, **MW043682**, **MW043678**, **MW043656**,  **MW043574** *A. italiana* from *C. subsubulata*, New Zealand  **MW043676**, **MW043670**, **MW043679**, **MW043583**, **MW043661** *A. italiana* from *C. capitellata*, New Zealand  **MW043669** *A. italiana* from *C. tenerrima*, New Zealand  **MW043668** *A. italiana* from *C. confusa*, New Zealand  **MW043677** *A. italiana* from *C. krempelhuberi*, New Zealand  **MW043662** *A. italiana* from *C. imbricata*, New Zealand  **MW043551** *A. italiana* from *C. cf. glebosa*, New Zealand  **MW043666** *A. italiana* from *C. darwinii*, New Zealand  **MW043615** *A. italiana* from *C. pleurota*, New Zealand  **MW043592** *A. italiana* from *C. ustulata*, New Zealand  **MW043631** *A. italiana* from *C. mawsonii*, New Zealand  **MW043584** *A. italiana* from *C. asperula*, New Zealand  **MW043649** *A. italiana* from *C. merochlorophaea*, Netherlands  **MW043648** *A. italiana* from *C. callosa*, Netherlands  **MW043495** *A. italiana* from *C. stereoclada*, Portugal, Azores  **MW043684** *A. italiana* from *C. foliacea*, Portugal, Azores  **MW043565** *A. italiana* from *C. capitellata*, Australia  **MW043560** *A. italiana* from *C. rappii s. lat*., Australia |
| Haplotype 170 | **MW043550** *A. magna* from *C. decorticata*, USA, Alaska |
| Haplotype 171 | **MW043580** *A. irregularis* from *C. nipponica*, USA, Alaska |
| Haplotype 172 | **MW043548** *A. magna* from *C. umbricola*, USA, Alaska |
| Haplotype 173 | **MW043608** *A. pseudoirregularis* from *C. gracilis* subsp*. vulnerata*, USA, Alaska  **MW043605**, **MW043606** *A. pseudoirregularis* from *C. amaurocraea*, USA, Alaska |
| Haplotype 174 | **MW043488** *A. mediterranea* from *C. rangiformis,* Turkey  **MW043508** *A. mediterranea* from *C. rangiformis,* Sweden  **MW043708**, **MW043709**, **MW043711** *A. mediterranea* from *C. corsicana*, Spain  **MW043496** *A. mediterranea* from *C. subturgida*, Spain  **MW043513** *A. mediterranea* from *C. rei*, Spain  **MW043572** *A. mediterranea* from *C. ramulosa s. lat*., Spain  **MW043693** *A. mediterranea* from *C. didyma*, Portugal, Azores  **MW043689**, **MW043685** *A. mediterranea* from *C. foliacea*, Portugal, Azores  **MW043710** *A. mediterranea* from *C. corsicana*, Portugal  **MW043571** *A. mediterranea* from *C. ramulosa s. lat*., Greece  **MW043593** *A. mediterranea* from *C. diversa*, Greece  **MW043514**, **MW043512**, **MW043510** *A. mediterranea* from *C. rangiformis*, Greece  **MW043518**, **MW043519**, **MW043520** *A. mediterranea* from *C. rangiformis*,  Cape Verde |
| Haplotype 175 | **MW043538** *A. mediterranea* from *C. ramulosa s. lat.*, Turkey |
| Haplotype 176 | **MW043695** *A*. sp. clade StA3 from *C. intermediella*, Tanzania |
| Haplotype 177 | **MW043595** *A*. sp. clade I2 from *C. fenestralis*, Taiwan |
| Haplotype 178 | **MW043503** *A*. sp. clade 8 from *C. rei*, Sweden |
| Haplotype 179 | **MW043501** *A. woessiae* from *C. conista*, Spain,  **MW043502** *A. woessiae* from *C. rangiformis*, Portugal, Madeira,  **MW043506** *A. woessiae* from *C. rangiformis*, Netherlands |
| Haplotype 180 | **MW043498** *A. woessiae* from *C. conista*, Spain |
| Haplotype 181 | **MW043509** *A. mediterranea* from *C. rei*, USA, Minnesota |
| Haplotype 182 | **MW043494** *A. mediterranea* from *C. conista*, Spain |
| Haplotype 183 | **MW043515** *A*. sp. clade CL100 from *C. confusa*, South Africa |
| Haplotype 184 | **MW043700** *A*. sp. clade 9 from *C. strepsilis*, Scotland |
| Haplotype 185 | **MW043705** *A. italiana* from *C. ciliata var. ciliata*, Scotland |
| Haplotype 186 | **MW043707** *A. italiana* from *C. bellidiflora*, Scotland |
| Haplotype 187 | **MW043492** *A*. sp. clade 8 from *C. conista*, Russia |
| Haplotype 188 | **MW043604** *A*. sp. CL249 from *C. scotteri* , Russia |
| Haplotype 189 | **MW043530** *A. irregularis* from *C. cineracea*, Russia |
| Haplotype 190 | **MW043526** *A. irregularis* from *C. alinii*, Russia |
| Haplotype 191 | **MW043652** *A. irregularis* from *C. albonigra*, Russia  **MW043611** *A. irregularis* from *C. botrytis*, Finland  **MW043646** *A. irregularis* from *C. uliginosa*, Finland  **MW043557** *A. irregularis* from *C. labradorica*, Canada |
| Haplotype 192 | **MW043635** *A. magna* from *C. chlorophaea*, Russia |
| Haplotype 193 | **MW043634** *A. irregularis* from *C. botrytes*, Russia  **MW043632** *A. irregularis* from *C. crispata*, Russia  **MW043594** *A. irregularis* from *C. uncialis subsp. uncialis*, Japan  **MW043647** *A. irregularis* from *C. macrophylla*, Finland  **MW043578** *A. irregularis* from *C. uncialis subsp. uncialis*, Finland |
| Haplotype 194 | **MW043497** *A*. sp. clade 8 from *C. conista,* USA, Connecticut |
| Haplotype 195 | **MW043543** *A. irregularis* from *C. vulcani*, Russia |
| Haplotype 196 | **MW043532** *A. pseudoirregularis* from *C. granulans*, Russia |
| Haplotype 197 | **MW043602** *A. irregularis* from *C. kanewskii*, Russia |
| Haplotype 198 | **MW043555** *A*. sp. clade 8 from *C. macilenta*, New Caledonia |
| Haplotype 199 | **MW043527** *A. italiana* from *C. foliacea*, Portugal, Azores  **MW043505** *A. italiana* from *C. rei*, Norway  **MW043651** *A. italiana* from *C. rei*, Finland |
| Haplotype 200 | **MW043688** *A*. sp. clade MN082 from *C. subradiata*, Portugal, Azores |
| Haplotype 201 | **MW043692** *A*. sp. clade 9 from *C. didyma*, Portugal, Azores |
| Haplotype 202 | **MW043690** *A*. sp. clade MN082 from *C. nana*, Portugal, Azores |
| Haplotype 203 | **MW043533** *A. glomerata* from *C. krogiana*, Norway |
| Haplotype 204 | **MW043552** *A*. sp. clade 9 from *C. medusina*, New Zealand  **MW043553** *A*. sp. clade 9 from *C. medusina*, Madagascar  **MW043559** *A*. sp. clade 9 from *C. ceratophyllina*, France, Reunion  **MW043567** *A*. sp. clade 9 from *C. coccifera s. lat.*, France, Reunion  **MW043598** *A*. sp. clade 9 from *C. didyma*, France, Reunion  **MW043516** *A*. sp. clade 9 from *C. mauritiana*, Cameroon |
| Haplotype 205 | **MW043671** *A. italiana* from *C. weymouthii*, New Zealand |
| Haplotype 206 | **MW043545** *A*. sp. clade CL100 from *C. imbricata*, New Zealand |
| Haplotype 207 | **MW043525** *A. italiana* from *C. fruticulosa*, New Zealand |
| Haplotype 208 | **MW043680** *A. italiana* from *C. subsubulata*, New Zealand |
| Haplotype 209 | **MW043667** *A. italiana* from *C. confusa*, New Zealand |
| Haplotype 210 | **MW043626** *A. italiana* from *C. neozelandica*, New Zealand |
| Haplotype 211 | **MW043686** *A*. sp. clade CL8 from *C. pyxidata s. lat.* , New Zealand |
| Haplotype 212 | **MW043555** *A. italiana* from *C. macilenta*, New Caledonia  **MW043554** *A. italiana* from *C. borbonica*, New Caledonia  **MW043556** *A. italiana* from *C. corymbescens*, New Caledonia  **MW043558** *A. italiana* from *C. confusa*, New Caledonia |
| Haplotype 213 | **MW043640** *A.glomerata* from *C. monomorpha, Netherlands* |
| Haplotype 214 | **MW043524** *A*. sp. clade 9 from *C. candelabrum s. lat*., Madagascar  **MW043517** *A*. sp. clade 9 from *C. camerunensis*, Cameroon |
| Haplotype 215 | **MW043489** *A. mediterranea* from *C. rangiformis*, Iran |
| Haplotype 216 | **MW043683** *A*. sp. clade CL79 from *C. didyma s. lat.,* Indonesia |
| Haplotype 217 | **MW043591** *A. mediterranea* from *C. crispata s. lat*., Greece |
| Haplotype 218 | **MW043581** *A*. *mediterranea* from C. glauca, Greece  **MW043590** *A*. *mediterranea* from *C. cryptochlorophaea*, Greece |
| Haplotype 219 | **MW043579** *A*. *italiana* from *C. graeca*, Greece |
| Haplotype 220 | **MW043569** *A*. sp. clade 9 from *C. mauritiana*, France, Reunion |
| Haplotype 221 | **MW043566** *A*. sp. clade MN082 from *C. ceratophylla*, France, Reunion |
| Haplotype 222 | **MW043568** *A*. sp. clade CL190 from *C. pachycladodes*, France, Reunion |
| Haplotype 223 | **MW043522** *A*. sp. clade 9 from *C. peltasta*, France, Reunion |
| Haplotype 224 | **MW043549** *A*. sp. clade 9 from *C. varians*, France, Reunion  **MW043582** *A*. sp. clade 9 from C. sp., France, Reunion |
| Haplotype 225 | **MW043529** *A*. sp. clade 9 from *C. ceratophyllina*, France, Reunion |
| Haplotype 226 | **MW043542** *A*. sp. clade 9 from *C. subreticulata*, France, Reunion |
| Haplotype 227 | **MW043601** *A. mediterranea* from *C.confusa*, France, Reunion |
| Haplotype 228 | **MW043599** *A*. sp. clade 9 from *C. gigantea s. lat.*, France, Reunion |
| Haplotype 229 | **MW043597** *A*. sp. clade 9 from *C. leptoclada*, France, Reunion |
| Haplotype 230 | **MW043585** *A*. sp. clade 9 from *C. centrophora*, France, Reunion |
| Haplotype 231 | **MW043600** *A*. sp. clade 9 from *C. cf. cinereorubens*, France, Reunion |
| Haplotype 232 | **MW043547** *A. glomerata* from *C. phyllophora*, France |
| Haplotype 233 | **MW043487** *A. glomerata* from *C. cornuta*, Finland |
| Haplotype 234 | **MW043491** *A*. sp. clade 8 from *C. rangiformis*, Finland |
| Haplotype 235 | **MW043610** *A. friedlii* from *C. fimbriata*, Finland |
| Haplotype 236 | **MW043541** *A. italiana* from *C. subcervicornis*, Faroe Island |
| Haplotype 237 | **MW043544** *A*. sp clade StA3 from *C. polydactyla*, Denmark |
| Haplotype 238 | **MW043654** *A*. sp. clade CL345 from *C. ramulosa s. lat*., Denmark |
| Haplotype 239 | **MW043659** *A.* sp. clade S1 from *C. arcuata*, Costa Rica |
| Haplotype 240 | **MW043657** *A*. sp. clade 9 from *C. dactylota*, Costa Rica  **MW043658** *A*. sp. clade 9 from *C. subdelicatula*, Costa Rica  **MW043645** *A*. sp. clade 9 from *C. farinophylla*, Bolivia  **MW043616** *A*. sp. clade 9 from *C. confusa*, Bolivia  **MW043621** *A*. sp. clade 9 from *C. compressa*, Bolivia |
| Haplotype 241 | **MW043655** *A*. sp. clade StA1 *C. isabellina*, Costa Rica |
| Haplotype 242 | **MW043665** *A*. sp. clade S1 from *C. andesita*, Costa Rica |
| Haplotype 243 | **MW043660** *A*. sp. clade S1 from *C. cortilaginea s. lat*., Costa Rica |
| Haplotype 244 | **MW043699** *A. italiana* from *C. lepidophora*, Chile |
| Haplotype 245 | **MW043499** *A. lobophora* from *C. rei*, Canada |
| Haplotype 246 | **MW043507** *A. lobophora* from *C. rei*, Canada |
| Haplotype 247 | **MW043663** *A*. *irregularis* from *C. ecmocyna subsp. occidentalis*, Canada |
| Haplotype 248 | **MW043620** *A*. sp. clade P2 from *C. calycantha*, Bolivia  **MW043644** *A*. sp. clade P2 from *C. mexicana*, Bolivia |
| Haplotype 249 | **MW043531** *A*. sp. clade S1 from *C. dactylota*, Bolivia |
| Haplotype 250 | **MW043642** *A*. sp. clade 9 from *C. solida*, Bolivia |
| Haplotype 251 | **MW043623** *A*. sp. clade StA1 from *C. melanopoda*, Bolivia |
| Haplotype 252 | **MW043613** *A*. sp. clade S1 from *C. meridensis*, Bolivia  **MW043627** *A*. sp. clade S1 from *C. diversa*, Bolivia |
| Haplotype 253 | **MW043617** *A*. sp. clade 9 from *C. calycantha*, Bolivia |
| Haplotype 254 | **MW043618** *A*. sp. clade P2 from *C. kriegeri*, Bolivia |
| Haplotype 255 | **MW043614** *A*. sp. clade StA1 from *C. leprocephala*, Bolivia  **MW043641** *A*. sp. clade StA1 from *C. cartilaginea*, Bolivia |
| Haplotype 256 | **MW043619** *A*. sp. clade 9 from *C. vescula*, Bolivia |
| Haplotype 257 | **MW043625** *A*. sp. clade StA1 from *C. leprocephala*, Bolivia |
| Haplotype 258 | **MW043624** *A*. sp. clade StA1 from *C. leprocephala*, Bolivia |
| Haplotype 259 | **MW043643** *A*. sp. clade S1 from *C. microscypha*, Bolivia |
| Haplotype 260 | **MW043628** *A*. sp. clade 9 from *C. granulosa*, Bolivia |
| Haplotype 261 | **MW043630** *A*. sp. clade P2 from *C. subsquamosa*, Bolivia |
| Haplotype 262 | **MW043629** *A*. sp. clade 8 from *C. pocillum s. lat.,* Bolivia |
| Haplotype 263 | **MW043637** *A*. sp. clade 9 from *C. pityrophylla*, Bolivia |
| Haplotype 264 | **MW043622** *A*. sp. clade StA1 from *C. isabellina*, Bolivia |
| Haplotype 265 | **MW043570** *A*. *italiana* from *C. rigida*, Australia |
| Haplotype 266 | **MW043564** *A*. sp. clade CL183 from *C. rappii s. lat.,* Australia |
| Haplotype 267 | **MW043561** *A. italiana* from *C. rappii s. lat.,* Australia |
| Haplotype 268 | **Kukwa-15135** *Asterochloris* sp. clade Bol 3 from *C*. *subradiata*, Bolivia |

Table S12. List of haplotypes showing relationships between ITS rDNA sequences of *Asterochloris* associating with *Lepraria* spp. Sequences from tropical region are marked in green.

| Haplotype | List of samples |
| --- | --- |
| Haplotype 1 | **Kukwa 14695** *A*. sp. clade Bol 2 from *L*. aff. *congesta*, La Paz 1 |
| Haplotype 2 | **Kukwa 14831** *A*. sp. clade Bol 2 from *L. congesta*, La Paz 1 |
| Haplotype 3 | **Kukwa 15186** *A*. sp. clade Bol 2 from *L. congesta*, Cochabamba 1,  **Kukwa 14827a** *A*. sp. clade Bol 2 from *L. congesta*, La Paz 1 |
| Haplotype 4 | **Kukwa 16204** *A*. sp. Bol 1 from *L. harrisiana*, Chuquisaca 2,  **Kukwa 16858, Kukwa 16831** *A*. sp. Bol 1 from *L. impossibilis*, Tarija 2,  **Kukwa 16584** *A*. sp. Bol 1 from *L. impossibilis*, Tarija 1 |
| Haplotype 5 | **Kukwa 16829** *A*. sp. clade A14 from *L*. cf. *cryptovouauxii*, Tarija 2 |
| Haplotype 6 | **Kukwa 16907** *A*. sp. clade A14 from *L. pallida*, Chuquisaca 2 |
| Haplotype 7 | **Kukwa 16941** *A*. sp. clade MN082 from *L. sipmaniana*, Tarija 2,  **Kukwa 16915b** *A*. sp. clade MN082 from *L. sipmaniana*, Tarija 1 |
| Haplotype 8 | **Kukwa 18069** *A. friedlii* from *L. achariana*, Cochabamba 1 |
| Haplotype 9 | **Kukwa** **19459** *A. friedlii* from *L. finkii*, Santa Cruz 1,  **AM905995** *A. friedlii* from *L. caesioalba*, Slovakia,  **EU008659** *A. friedlii* from *L. cupressicola*, China,  **EU008661**, **EU008662** *A. friedlii* from *L. caesiella*, USA,  **EU008663**, **EU008666** *A. friedlii* from *L. caesioalba*, USA,  **EU008668** *A. friedlii* from *L. incana*, USA,  **EU008674**, **EU008676**, **EU008677**, **EU008679**, **EU008680** *A. friedlii* from *L. finkii*, USA,  **FN556034** *A. friedlii* from *L. rigidula*, Czech Republic |
| Haplotype 10 | **Kukwa** **19468** *A*. sp. clade L54 from *L.* aff*. hodkinsoniana*, Santa Cruz 1 |
| Haplotype 11 | **Kukwa** **19499** *A*. sp. clade Bol 6 from *L*. *impossibilis,* Santa Cruz 1 |
| Haplotype 12 | **Kukwa** **14848a** *A*. sp. clade A6 from *L. cryptovouauxii*, La Paz 2 |
| Haplotype 13 | **AM900492**, **AM906015** *A. woessiae* from *L. borealis*, Bulgaria,  **AM906014** *A. woessiae* from *L. caesioalba*, Czech Republic |
| Haplotype 14 | **AM905992** *A. echinata* from *L. rigidula*, Czech Republic,  **FN556029** *A. echinata* from *L. granulata*, Bulgaria |
| Haplotype 15 | **AM905994** *A. friedlii* from *L*. cf. *caesioalba*, Slovakia |
| Haplotype 16 | **AM905997**, **KP318664**, **FM955668** *A. gaertneri* from *L. rigidula*, Czech Republic,  **FN556037** *A. gaertneri* from *L. incana*, Czech Republic,  **FN556038** *A. gaertneri* from *L. membranacea*, Czech Republic |
| Haplotype 17 | **AM906002**, **AM906005** *A. leprarii* from *L. neglecta*, Czech Republic,  **AM906003**, **AM906004** *A. leprarii* from *L. caesioalba,* Czech Republic |
| Haplotype 18 | **AM906008**, **AM906009** *A. lobophora* from *L. caesioalba*, Czech Republic,  **AM906010** *A. lobophora* from *L. alpina*, Czech Republic,  **AM906007** *A. lobophora* from *L*. cf. *caesioalba*, Czech Republic |
| Haplotype 19 | **DQ229877** *A. friedlii* from *L. finkii*, USA: Wisconsin |
| Haplotype 20 | **DQ229878** *A. lobophora* from *L. finkii*, USA:Wisconsin |
| Haplotype 21 | **FJ406576** *A*. sp. clade A9 from *L. borealis*, Antarctica |
| Haplotype 22 | **FM955667** *A. echinata* from *L. caesioalba*, Czech Republic,  **FN556026** *A. echinata* from *L. alpina*, Spain |
| Haplotype 23 | **FM955669** *A. gaertneri* from *L. rigidula,* Czech Republic |
| Haplotype 24 | **FN556031** *A*. sp. clade A4 from *L. rigidula*, Czech Republic |
| Haplotype 25 | **FN556035** *A*. *stereocaulonicola* from *L. alpina*, Spain |
| Haplotype 26 | **FN556042** *A*. sp. clade A11 from *L. caesioalba*, USA: California |
| Haplotype 27 | **FN556044** *A. lobophora* from *L. borealis*, Czech Republic |
| Haplotype 28 | **EU008664**, **EU008665** *A. friedlii* from *L. caesioalba*, USA,  **EU008678** *A. friedlii* from *L. finkii*, USA |
| Haplotype 29 | **EU008681** *A*. sp. clade L55 from *L. yunnaniana*, Costa Rica |
| Haplotype 30 | **EU008683** *A. mediterranea* from *L. nylanderiana*, Italy |
| Haplotype 31 | **AM906013**, **AM900491** *A. phycobiontica* from *L. neglecta*, Ukraine,  **FN556023** *A. phycobiontica* from *L. alpin*a, Czech Republic,  **FN556024** *A. phycobiontica* from *L. caesioalba*, Czech Republic |
| Haplotype 32 | **AM906006** *A. lobophora* from *L. caesioalba*, Czech Republic |
| Haplotype 33 | **AM905996** *A. friedlii* from *L. caesioalba*, Romania |
| Haplotype 34 | **FN556027** *A. echinata* from *L. caesioalba*, Slovakia |
| Haplotype 35 | **FN556028** *A. echinata* from *L. caesioalba*, Spain |
| Haplotype 36 | **FN556033** *A*. sp. clade A6 from *L. crassissima*, Czech Republic |
| Haplotype 37 | **FN556045** *A. lobophora* from *L. caesioalba*, Czech Republic |
| Haplotype 38 | **KP318670** *A. friedlii* from *L. elobat*a, Slovakia |
| Haplotype 39 | **FN556030** *A*. sp. clade A4 from *L. caesioalba*, Czech Republic,  **FN556032** *A*. sp. clade A4 from *L. rigidula*, Czech Republic |
| Haplotype 40 | **FN556036** *A*. *stereocaulonicola* from *L. caesioalba*, Czech Republic |
| Haplotype 41 | **FN556039** *A*. sp. clade A11 from *L. borealis*, USA: California |
| Haplotype 42 | **FN556040** *A*. sp. clade A11 from *L. caesioalba*, USA: California |
| Haplotype 43 | **FN556041** *A.* sp. clade A11 from *L. caesioalba*, USA: California |
| Haplotype 44 | **Kukwa 16828** *A*. sp. Bol 2 from *L. impossibilis,* Tarija 2, |

Table S13. List of haplotypes showing relationships, between ITS rDNA sequences of *Asterochloris* from lichen forming fungi, representing cosmopolitan distribution pattern. Sequences from tropical region are marked in green.

| Haplotype  number | Sample ID |
| --- | --- |
| Haplotype 1 | **FM945347** *A. irregularis* from *Cladonia arbuscula*, Czech Republic,  **MH415358**, **MH415360**, **MH415361** *A. irregularis* from S*tereocaulon alpinum*, Greenland,  **MH415368** *A. irregularis* from *Stereocaulon tomentosum/sasakii*, USA: Alaska,  **MH415394** *A. irregularis* from *Stereocaulon alpinum*, Finland,  **KT989933** *A. irregularis* from *Cladonia coccifera,* Czech Republic,  **KT989930**, **KT989929** *A. irregularis* from *Cladonia coccifera,* Finland,  **AF345409** *A. irregularis* from *Cladonia coccifera*, Russia,  **AM906000** *A. irregularis* from *Cladonia arbuscula*, Slovakia |
| Haplotype 2 | **FM945351**, **FM945352**, **FM945353** *A. glomerata* from *Cladonia coccifera*, Czech Republic,  **DQ530181** *A. glomerata* from *Cladonia pyxidata,* Canada |
| Haplotype 3 | **HE803028** *A. glomerata* from *Cladonia coniocraea*, Nepal |
| Haplotype 4 | **HE803035**, **HE803036** *A*. sp. clade I2 from *Cladonia furcata*, India |
| Haplotype 5 | **HE803037** *A*. sp. clade I2 from *Cladonia* *furcata*, India |
| Haplotype 6 | **KP314492** *A. erici* from *Cladonia pocillum*, Svalbard |
| Haplotype 7 | **KP314498** *A*. sp clade A19 from *Cladonia pocillum*, Svalbard |
| Haplotype 8 | **KP314503** *A. erici* from *Cladonia pocillum*, Svalbard |
| Haplotype 9 | **KP314523** *A*. *magna* from *Cladonia pocillum*, Svalbard |
| Haplotype 10 | **KP314558** *A*. *magna* from *Cladonia pocillum*, Svalbard |
| Haplotype 11 | **KP314582** *A*. *magna* from *Cladonia pocillum*, Svalbard |
| Haplotype 12 | **KP314598** *A. erici* from *Cladonia pocillum*, Svalbard |
| Haplotype 13 | **KP314604** *A*. *magna* from *Cladonia pocillum*, Svalbard |
| Haplotype 14 | **KP314633** *A. erici* from *Cladonia pocillum*, Svalbard |
| Haplotype 15 | **KP314635** *A*. *magna* from *Cladonia pocillum*, Svalbard |
| Haplotype 16 | **KP314665** *A*. sp clade A19 from *Cladonia pocillum*, Svalbard |
| Haplotype 17 | **MH415323** *A*. *pseudoirregularis* from *Stereocaulon tomentosum/sasakii*, USA: Alaska |
| Haplotype 18 | **MH415409** *A*. *pseudoirregularis* from *Stereocaulon alpinum*, Canada,  **KT989936** *A*. *pseudoirregularis* from *Cladonia coccifera*, Norway |
| Haplotype 19 | **MH415229** *A*. sp. clade StA8 from *Stereocaulon* *myriocarpum*, Costa Rica |
| Haplotype 20 | **MH415287** *A*. sp. clade StA8 from *Stereocaulon myriocarpum*, Venezuela |
| Haplotype 21 | **MH415288** *A*. sp. clade StA8 from *Stereocaulon myriocarpum*, Venezuela |
| Haplotype 22 | **MH415294** *A*. sp. clade StA8 from *Stereocaulon* *myriocarpum*, Venezuela |
| Haplotype 23 | **Kukwa 14827** *A*. sp. clade Bol 7 from *Stereocaulon alpinum*, La Paz 1 |
| Haplotype 24 | **Kukwa 15040** *A*. sp. clade 12 from *Cladonia chlorophaea*, Cochabamba 1,  **HE803050** A. sp. clade 12 from *Cladonia pyxidata*, India |
| Haplotype 25 | **Kukwa 15044** *A*. sp. clade P2 from *Cladonia furcata*, Cochabamba 1 |
| Haplotype 26 | **Kukwa 16553** *A*. sp. clade Bol 5 from *Cladonia furcata*, Tarija 1 |
| Haplotype 27 | **UGDA-L 18613** *A*. sp. clade P2 from *Stereocaulon myriocarpum*, La Paz 4,  **UGDA-L 19021** *A*. sp. clade P2 from *Cladonia arbuscula*, Santa Cruz 1 |
| Haplotype 28 | **UGDA-L 18963** *A*. sp. clade StA5 from *Cladonia pocillum*, La Paz 5,  **MH415221**, **MH415222**, **MH415224**, **MH415225** *A*. sp. clade StA5 from S*tereocaulon* *alpinum,* Georgia,  **MH415366** *A*. sp. clade StA5 from *Stereocaulon alpinum*, Austria |
| Haplotype 29 | **UGDA-L** **19009** *A*. sp. clade 9 from *Cladonia arcuata*, Santa Cruz 2 |
| Haplotype 30 | **Kukwa** **19459** *A. friedlii* sp. from *Lepraria finkii*, Santa Cruz 1 |
| Haplotype 31 | **UGDA-L 25173** *A*. sp. clade S1 from *Stereocaulon tomentosum*, Santa Cruz 1 |
| Haplotype 32 | **AF345431** *A*. sp. clade 8 from *Cladonia chlorophaea*, Canada,  **KT989918**, KT989917 *A*. sp. clade 8 from *Cladonia coccifera*, Czech Republic,  **KT989916** *A*. sp. clade 8 from *Cladonia coccifera*, Austria,  **DQ530188** *A*. sp. clade 8 from *Cladonia chlorophaea*, Canada |
| Haplotype 33 | **DQ229877** *A*. *friedlii* from *Lepraria finkii*, USA: Wisconsin |
| Haplotype 34 | **DQ229878** *A. lobophora* from *Lepraria finkii*, USA: Wisconsin |
| Haplotype 35 | **FM945354** *A. lobophora* from *Cladonia coniocraea*, Czech Republic,  **FM945373** *A. lobophora* from *Cladonia pyxidata*, Czech Republic,  **FM945391** *A. lobophora* from *Stereocaulon tomentosum*, Slovakia,  **MH415297**, **MH415298**, **MH415299** *A. lobophora* from *Stereocaulon alpinum*, Georgia,  **DQ530184** *A. lobophora* from *Cladonia pocillum*, Canada,  **DQ530179** *A. lobophora* from *Cladonia coniocraea*, Canada,  **KT989907**, **KP318674** *A. lobophora* from *Cladonia coccifera,* Czech Republic |
| Haplotype 36 | **FM945360**, **FM945361**, **FM945362** *A*. sp. clade 12 from *Cladonia furcata*, Czech Republic,  **FM945374**, **FM945375** *A*. sp. clade 12 from *Cladonia pyxidata*, Czech Republic,  **DQ530190** *A*. sp. clade 12 from *Cladonia pocillum*, Canada |
| Haplotype 37 | **HE803030** *A*. sp. clade I1 form *Cladonia furcata*, India |
| Haplotype 38 | **HE803031** *A*. sp. clade I1 from *Cladonia pyxidata*, India  **HE803032** *A*. sp. clade I1 from *Cladonia furcata*, India |
| Haplotype 39 | **HE803039** *A*. sp. clade 9 from *Cladonia coniocraea*, Nepal |
| Haplotype 40 | **KX051235** *A. sajongensis* from *Cladonia pyxidata*, Antarctica |
| Haplotype 41 | **MH415217** *A. italiana* from *Stereocaulon alpinum*, South Argentina |
| Haplotype 42 | **MH415257**, **MH415258** *A*. *antarctica* from *Stereocaulon alpinum*, Iceland |
| Haplotype 43 | **MH415334** *A. phycobiontica* from *Stereocaulon alpinum*, Austria |
| Haplotype 44 | **MH415357** *A*. sp. clade StA4 from *Stereocaulon alpinum*, Greenland  **KT989905** *A*. sp. clade StA4 from *Cladonia coccifera,* Czech Republic |
| Haplotype 45 | **MH415364** *A*. sp clade StA4 from *Stereocaulon alpinum*, Greenland |
| Haplotype 46 | **MH415383** *A. woessiae* from *Stereocaulon alpinum*, Denmark  **KP318669** *A. woessiae* from *Cladonia coccifera*, Spain |
| Haplotype 47 | **MH415410** *A*. sp. clade StA5 from *Stereocaulon* *alpinum*, Canada |
| Haplotype 48 | **Kukwa 14831** *A*. sp. clade Bol 2 from *Lepraria* *congesta*, La Paz 1 |
| Haplotype 49 | **Kukwa 15186** *A*. sp. clade Bol 2 from *Lepraria congesta*, Cochabamba 1 |
| Haplotype 50 | **AF345436** *A*. sp. clade 12 from *Cladonia pyxidata*, Italy |
| Haplotype 51 | **MW043686** *A*. sp. clade 9 from *Cladonia pyxidata s. lat*., New Zealand |
| Haplotype 52 | **MW043629** *A*. sp. clade 8 from *Cladonia pocillum s. lat*., Bolivia |
| Haplotype 53 | **DQ530191** *A*. *leprarii* from *Cladonia pocillum*, Canada |
| Haplotype 54 | **AF345429** *A*. sp. clade 8 from *Cladonia furcata*, USA: Virginia |
| Haplotype 55 | **KT989901** *A. italiana* from *Cladonia coccifera*, Denmark*,*  **KT989904** *A. italiana* from *Cladonia coccifera*, Norway*,*  **KT989896**, **KT989897** *A. italiana* from *Cladonia coccifera, UK,*  **KT989890** *A. italiana* from *Cladonia coccifera,* Czech Republic |
| Haplotype 56 | KT989888 *A. italiana* from *Cladonia coccifera,* Czech Republic |
| Haplotype 57 | **KT989908**, **KT989909**, **KT989910**, **KT989914** *A.* aff. *italiana from Cladonia coccifera,* Czech Republic,  **KT989911** *A.* aff. *italiana from Cladonia coccifera*, Austria |
| Haplotype 58 | **KT989922** *A*. *stereocaulonicola* from *Cladonia coccifera,* Czech Republic |
| Haplotype 59 | **MW043567** *A*. sp. clade 9 from *Cladonia coccifera s. lat*., France, Reunion |
| Haplotype 60 | **KT989919**, **KT989921** A. sp. clade StA3 from *Cladonia coccifera*, Austria  **KT989920** *A*. sp. clade StA3 from *Cladonia coccifera,* Czech Republic |
| Haplotype 61 | **KT989915** *A*. *antarctica* from *Cladonia coccifera*, Austria |
| Haplotype 62 | **MW043635** *A. magna* from *Cladonia chlorophaea,* Russia |

Table S14. List of haplotypes showing relationships, between ITS rDNA sequences of *Asterochloris* from lichen forming fungi, representing Neotropical distribution pattern.

| Haplotype number | Sample ID |
| --- | --- |
| Haplotype 1 | **Kukwa 16168** *A*. sp. clade MN082 form *Cladonia* aff. *ahtii*, Chuquisaca 2,  **Kukwa 16909** *A.* sp. clade MN082 from *Cladonia* aff. *ahtii*, Chuquisaca 2,  **Kukwa 14785** *A.* sp. clade MN082 from *Cladonia ceratophylla*, La Paz 1,  **Kukwa 18470** *A.* sp. clade MN082 from *Cladonia ceratophylla*, Santa Cruz 1 |
| Haplotype 2 | **UGDA-L 19009** *A*. sp. clade 9 from *Cladonia arcuata*, Santa Cruz 2 |
| Haplotype 3 | **UGDA-L 18961** *A*. *mediterranea* from *Cladonia calycantha*, La Paz 3 |
| Haplotype 4 | **Kukwa 14886** *A*. sp. clade P2 from *Cladonia calycantha*, La Paz 2 |
| Haplotype 5 | **AY842277** *A*. sp. from *Cladonia crinita*, Brazil |
| Haplotype 6 | **UGDA-L 19015** *A*. sp. clade 9 from *Cladonia dactylota*, Santa Cruz 1,  **Kukwa 16205** *A.* sp. clade 9 from *Cladonia granulosa*, Chuquisaca 2 |
| Haplotype 7 | **AY842278** *A*. sp. from *Cladonia fissidens*, Brazil |
| Haplotype 8 | **Kukwa 14848a** *A*. sp. clade A6 from *Lepraria cryptovouauxii*, La Paz 2 |
| Haplotype 9 | **Kukwa 19468** *A*. sp. clade L54 from *Lepraria* aff. *hodkinsoniana*, Santa Cruz 1 |
| Haplotype 10 | **Kukwa 16831**, **Kukwa 16858** *A*. sp. clade Bol 1 from *Lepraria impossibilis*, Tarija 2,  **Kukwa 16584** *A.* sp. clade Bol 1 from *Lepraria impossibilis*, Tarija 1 |
| Haplotype 11 | **UGDA-L 18555** *A*. sp. clade Bol 4 from *Stereocaulon microcarpum*, La Paz 3 |
| Haplotype 12 | **UGDA-L 18566** *A*. *mediterranea* from *Stereocaulon pachycephalum*, La Paz 4 |
| Haplotype 13 | **UGDA-L 18992** *A*. sp. clade StA1 from *Cladonia isabellina*, La Paz 4 |
| Haplotype 14 | **Kukwa 14684** *A*. sp. clade StA1 from *Cladonia andesita*, La Paz 1 |
| Haplotype 15 | **UGDA-L 18556** *A*. sp. clade Bol 8 from *Lepraria achariana*, Santa Cruz 1 |
| Haplotype 16 | **Kukwa 16829** *A*. sp. clade A14 from *Lepraria* cf. *cryptovouauxii*, Tarija 2 |
| Haplotype 17 | **Kukwa 16586** *A*. sp. clade Bol 3 from *Diploschistes* cf. *cinereocesius*, Tarija 1 |
| Haplotype 18 | **Kukwa-16828** *A*. sp. clade Bol 1 from *Lepraria impossibilis*, Tarija 2 |
| Haplotype 19 | **Kukwa-16907a** *A*. sp. clade Bol 9 from *Lepraria impossibilis*, Chuquisaca 2 |
| Haplotype 20 | **MW043665** *A*. sp. clade S1 from *Cladonia andesita*, Costa Rica |
| Haplotype 21 | **MW043659** *A*. sp. clade S1 from *Cladonia arcuata*, Costa Rica |
| Haplotype 22 | **MW043566** *A*. sp. clade MN082 form *Cladonia ceratophylla*, France, Reunion |
| Haplotype 23 | **MW043657** *A*. sp. clade 9 from *Cladonia dactylota*, Costa Rica |
| Haplotype 24 | **MW043655** *A*. sp. clade StA1 from *Cladonia isabellina*, Costa Rica |
| Haplotype 25 | **MW043628** *A*. sp. clade 9 from *Cladonia granulosa*, Bolivia |
| Haplotype 26 | **MW043620** *A*. sp. clade P2 from *Cladonia calycantha*, Bolivia |
| Haplotype 27 | **MW043617** *A*. sp. clade 9 from *Cladonia calycantha*, Bolivia |
| Haplotype 28 | **MW043531** *A*. sp. clade S1 from *Cladonia dactylota*, Bolivia |
| Haplotype 29 | **MW043622** *A*. sp. clade StA1 from *Cladonia isabellina*, Bolivia |

Table S15. List of haplotypes showing relationships, between ITS rDNA sequences of *Asterochloris* from lichen forming fungi, representing Pantropical distribution pattern.

| Haplotype number | Sample ID |
| --- | --- |
| Haplotype 1 | **UGDA-L 18987** *A*. sp. clade P2 from *Cadonia melanopoda*, La Paz 4,  **Kukwa 16911** *A*. sp. clade P2 from *Cladia aggregata*, Chuquisaca 2,  **UGDA-L 19004** *A*. sp. clade P2 from *Cladonia didyma*, La Paz 4 |
| Haplotype 2 | **AY842279** *A*. sp. clade 9 from *Cladonia confusa*, Brazil |
| Haplotype 3 | **UGDA-L 18999** *A*. sp. clade S1 from *Cladonia didyma*, La Paz 2 |
| Haplotype 4 | **HE803045**, **HE803046**, **HE803047** *A*. sp. clade 9 from *Cladonia fruticulosa*, India |
| Haplotype 5 | **Kukwa 15987**, **Kukwa 15988** *A*. sp. clade Bol 2 from *Diploschistes hypoleucus*,  Chuquisaca 1 |
| Haplotype 6 | **Kukwa 16204** *A*. sp. clade Bol 1 from *Lepraria harrisiana*, Chuquisaca 2 |
| Haplotype 7 | **Kukwa 16907** *A*. sp. clade A14 from *Lepraria pallida*, Chuquisaca 2 |
| Haplotype 8 | **Kukwa 16915b** *A*. sp. clade MN082 from *Lepraria sipmaniana*, Tarija 1,  **Kukwa 16941** *A*. sp. clade MN082 from *Lepraria sipmaniana*, Tarija 2 |
| Haplotype 9 | **AF345437** *A*. sp. clade StA7 from *Cladia aggregata*, Chile |
| Haplotype 10 | **HE803048** A. sp. clade 9 from *Cladonia praetermissa*, India |
| Haplotype 11 | **HE803042** *A*. sp. clade 9 from *Cladonia delavayi*, India |
| Haplotype 12 | **HE803040** *A*. sp. clade 9 from *Cladonia delavayi*, India |
| Haplotype 13 | **Kukwa 15030b** *A*. sp. clade StA1 from *Cladonia confusa*, Cochabamba 1 |
| Haplotype 14 | **Kukwa 16621** *A*. sp. clade StA1 from *Cladonia confusa*, Tarija 1 |
| Haplotype 15 | **MW043515** *A*. sp. clade CL100 from *Cladonia confusa*, South Africa |
| Haplotype 16 | **MW043668** *A*. *italiana* from *Cladonia confusa*, New Zealand |
| Haplotype 17 | **MW043667** *A*. *italiana* from *Cladonia confusa*, New Zealand |
| Haplotype 18 | **MW043601** *A. mediterranea* from *Cladonia confusa*, France, Reunion |
| Haplotype 19 | **MW043558** *A. italiana* from *Cladonia confusa*, New Caledonia |
| Haplotype 20 | **JN990653** *A*. sp. clade 9 from *Cladonia confusa*, Brasil |
| Haplotype 21 | **JN990652** *A*. sp. clade 9 from *Cladonia confusa*, Brasil |
| Haplotype 21 | **JN990651** *A*. sp. clade 9 from *Cladonia confusa*, Brasil |
| Haplotype 22 | **EU008658** *A*. sp. clade 9 from *Cladia aggregata*, Costa Rica |
| Haplotype 23 | **AF345415** *A*. sp. clade MN082 from *Cladonia didyma*, USA: Alabama |
| Haplotype 24 | **JN990659** *A*. sp. clade 9 from *Cladonia didyma*, Brasil |
| Haplotype 25 | **MW043683** *A*. sp. clade CL79 from *Cladonia didyma*, Indonesia |
| Haplotype 26 | **MW043693** *A. mediterranea* from *Cladonia didyma* , Portugal, Azores |
| Haplotype 27 | **MW043692** *A*. sp. clade 9 from *Cladonia didyma* , Portugal, Azores |
| Haplotype 28 | **MW043598** *A*. sp. clade 9 from *Cladonia didyma*, France, Reunion |
| Haplotype 29 | **MW043562** *A*. sp. clade MN082 from *Cladonia didyma*, USA, Hawaii |
| Haplotype 30 | **MW043525** *A. italiana* from *Cladonia fruticulosa*, New Zealand |
| Haplotype 31 | **MW043616** *A*. sp. clade 9 from *Cladonia confusa*, Bolivia,  **MW043621** *A*. sp. clade 9 from *Cladonia melanopoda*, Bolivia |
| Haplotype 32 | **MW043623** *A*. sp. clade StA1 from *Cladonia melanopoda*, Bolivia |

Table S16. Summary of data for haplotype networks.

Table S16. Summary of data for haplotype networks.

| Variable/dataset | *Stereocaulon* | *Cladonia* | *Lepraria* | Cosmopolitan | Neotropical | Pantropical |
| --- | --- | --- | --- | --- | --- | --- |
| Number of samples [N] | 200 | 630 | 82 | 116 | 35 | 40 |
| Number of mycobionts species [N] | 36 | 178 | 26 | 12 | 17 | 10 |
| Alignment length | 501 | 535 | 503 | 505 | 503 | 501 |
| Nucleotide diversity | 0.01700833 | 0.00231309 | 0.02065656 | 0.02515689 | 0.02037583 | 0.02252267 |
| Number of *Asterochloris* haplotypes [N] | 55 | 268 | 44 | 62 | 29 | 32 |
| Haplotype diversity | 0.28 | 0.42 | 0.54 | 0.53 | 0.83 | 0.80 |

Table S17. Summary of data for haplotype networks with division into tropical and temperate samples.

| Variable/dataset | *Stereocaulon* tropical | *Stereocaulon* temperate | *Cladonia* tropical | *Cladonia* temperate | *Lepraria*  tropical | *Lepraria*  temperate | Cosmopolitan tropical | Cosmopolitan temperate |
| --- | --- | --- | --- | --- | --- | --- | --- | --- |
| Numper of samples [N] | 46 | 151 | 210 | 407 | 20 | 61 | 32 | 83 |
| Number of mycobionts species [N] | 10 | 24 | 99 | 101 | 13 | 14 | 13 | 10 |
| Number of *Asterochloris* haplotypes [N] | 24 | 36 | 132 | 143 | 14 | 30 | 24 | 38 |
| Haplotype diversity | 0.52 | 0.24 | 0.63 | 0.35 | 0.70 | 0.49 | 0.75 | 0.46 |
